# Behavioral effects of visual stimuli in adult zebrafish using a novel eight-tank imaging system

**DOI:** 10.3389/fnbeh.2024.1320126

**Published:** 2024-03-11

**Authors:** Sayali V. Gore, Thaís Del Rosario Hernández, Robbert Creton

**Affiliations:** Department of Molecular Biology, Cell Biology, and Biochemistry, Brown University, Providence, RI, United States

**Keywords:** zebrafish, OMR, moving lines, optomotor response, visually guided behavior, high-throughput, zebrafish adults

## Abstract

**Introduction:**

Animals respond to various environmental cues. Animal behavior is complex, and behavior analysis can greatly help to understand brain function. Most of the available behavioral imaging setups are expensive, provide limited options for customization, and allow for behavioral imaging of one animal at a time.

**Methods:**

The current study takes advantage of adult zebrafish as a model organism to study behavior in a novel behavioral setup allowing one to concurrently image 8 adult zebrafish.

**Results:**

Our results indicate that adult zebrafish show a unique behavioral profile in response to visual stimuli such as moving lines. In the presence of moving lines, the fish spent more time exploring the tank and spent more time toward the edges of the tanks. In addition, the fish moved and oriented themselves against the direction of the moving lines, indicating a negative optomotor response (OMR). With repeated exposure to moving lines, we observed a reduced optomotor response in adult zebrafish.

**Discussion:**

Our behavioral setup is relatively inexpensive, provides flexibility in the presentation of various animated visual stimuli, and offers improved throughput for analyzing behavior in adult zebrafish. This behavioral setup shows promising potential to quantify various behavioral measures and opens new avenues to understand complex behaviors.

## Introduction

Zebrafish are known to show a plethora of behaviors from simple visually guided responses to complex social behaviors (Kalueff et al., 2013, 2014; Stewart et al., 2014). A fully characterized genome, availability of genetic mutant lines, and ease of molecular manipulations make zebrafish well-suited for studying neural processes underlying behavior in normal and disease models (Norton and Bally-Cuif, 2010; Choi et al., 2021; Soussi-Yanicostas, 2022). Zebrafish serve as an excellent model organism for studying genetics, physiology, and behavior (Norton and Bally-Cuif, 2010; Kalueff et al., 2014; Choi et al., 2021). While zebrafish larvae, due to their size, easy availability in large numbers, and relatively simple behaviors, are suitable models for conducting high-throughput screens (Richendrfer and Creton, 2013, 2018; Clift et al., 2014; Thorn et al., 2019; Edmister and Creton, 2022; Edmister et al., 2022a,b; Gore et al., 2023), adult zebrafish display a full repertoire of sophisticated behaviors making their behavioral characterization important for understanding neural underpinnings of complex behavior and to understand how behaviors evolve during development. Zebrafish larvae are extensively used to discover new drugs or repurpose existing drugs for treating age-related neurological disorders. With the availability of baseline behavioral studies available in larval stages, it is important to understand how zebrafish adults respond to the same/similar stimuli. Developing novel paradigms to study behavior in adult zebrafish is important to understand the effect of not only the normal aging brain but also of various models of neurological disorders to investigate the neural processes involved in neurological disorders.

Zebrafish embryos develop externally and use maternally supplied yolk as the source of food during the initial stages of development. As the yolk depletes, zebrafish larvae need to find food resources and show prey-capture movements and food-seeking behaviors (Neuhauss, 2010). From early developmental stages, zebrafish have evolved ways to respond to environmental stimuli by showing behaviors such as prey capture, orientation, and escape responses from predators. Zebrafish from as early as 70 h post-fertilization show visually mediated behaviors such as visual startle response, optokinetic response, and so on (Kimmel et al., 1974; Easter and Nicola, 1996; Portugues and Engert, 2009; Neuhauss, 2010). The behavioral progression to visual stimuli in zebrafish aligns well with the maturation of the nervous and visual systems (Neuhauss, 2010). Behavioral responses to visual stimuli are extremely suitable for studying behavior as various stimulus properties such as brightness, colors, contrast, frequency, etc. can be altered to study behavioral responses. To understand neural processes involved in visually guided behaviors, one needs to develop and optimize behavioral paradigms.

Quantification of neural function can be greatly enhanced by the characterization of behavior (Mathis and Mathis, 2020; Mathis, 2021). Animals respond to different stimuli present in the environment and often display complex behaviors. These complex behaviors can often be explained by quantifying multiple simple behavioral parameters. Accurate quantification of simple behaviors is extremely important for understanding complex behaviors (Pereira et al., 2020; Maree et al., 2023). Technological advances in image acquisition with high-quality video cameras and the presence of commercially available imaging equipment have helped with the quantification of behavior (Nath et al., 2019). Various studies have used zebrafish larvae as a model organism to study behavior in a 384-well high-throughput setting (Richendrfer and Creton, 2013; Clift et al., 2014; Edmister and Creton, 2022; Edmister et al., 2022a,b; Gore et al., 2023). However, there are limited reports of behavioral analysis of adult zebrafish in high-throughput settings (Stewart et al., 2015).

Compared to the behavioral studies in zebrafish larvae, the analysis of behavior in zebrafish adults is limited. In zebrafish adults, most of the studies focus on locomotor effects (DePasquale et al., 2022), social interactions (Buske and Gerlai, 2011; Green et al., 2012; Sykes et al., 2018), and cognitive behaviors (DePasquale et al., 2021). Many studies characterizing social behaviors image adult zebrafish in a relatively high-throughput fashion which involves using multiple animals in one tank and labeling individual animals (Buske and Gerlai, 2011; Green et al., 2012; Perez-Escudero et al., 2014; Sykes et al., 2018). Tracking multiple animals often requires using expensive setups and software to study behaviors. Many other complex behaviors such as anxiety, sleep, learning, etc. are also studied in zebrafish adults and use expensive imaging analysis systems that are often not flexible enough to accommodate the presentation of various stimuli. While most investigators image one tank at a time, one group was able to measure acoustic startle responses in 36 adult zebrafish using an array of 3.5 cm × 3.5 cm compartments (Bang et al., 2002). The current study describes a behavioral setup for imaging eight adult zebrafish simultaneously, using small tanks that allow for the analysis of multiple behaviors in response to animated visual stimuli. The imaging and analysis methods used in the study are open-source, inexpensive, and flexible enough to check responses to various stimuli. With highly characterized genetics, advances in molecular techniques, and sophisticated behavioral quantification tools, zebrafish adults can serve as an ideal model for understanding neural underpinnings of behavior during normal and diseased stages.

## Materials and methods

### Experimental animals

All experiments in the current study used adult *Danio rerio* (15 months old). The experiments were carried out in accordance with federal regulations and guidelines for the ethical and humane use of animals and were approved by Brown University’s Institutional Animal Care and Use Committee (Animal Welfare Assurance Number D16-00183). For the current study, a total of 16 adult wild-type *Danio rerio* (eight females and eight males) were used. The wild-type animals used for the study were genetically diverse strains acquired from Carolina Biological. The adult animals were maintained in a room separate from the imaging room in groups of approximately 40–50 per 30-gallon tank. The water in the tanks was recirculated in an automatic fashion and maintained at appropriate environmental conditions (Temperature 28°C, pH 7.2, and a 14:10-h light: dark cycle) suitable for the growth and maintenance of *Danio rerio*. The animals used for the study were checked routinely by animal care and were considered healthy, and devoid of pathogens or other abnormalities.

### Experimental design

The adult zebrafish (*n* = 16, eight males and eight females) were imaged during the same time window (1–5 pm) on different days in a balanced fashion. For example, four males and four females were imaged on each day. Behavioral analysis is sensitive to circadian fluctuations (Hurd et al., 1998; Barnard and Nolan, 2008) and so, imaging was performed in the 1–5 p.m. time window. Age-matched adult zebrafish were placed in white opaque tanks (Thomas Scientific Cat No. 1113H35, Length = 11.5 in, Width = 7.5 in, Height = 5 in) containing 1 L of water. Using automated methods for imaging, image analysis, and data processing allowed us to acquire robust reliable behavioral measures. The experimental design, the sample size of zebrafish adults, and the statistical analysis reported in the current study are in accordance with the ARRIVE (Animal Research: Reporting of *In Vivo* Experiments) guidelines.

### Experimental setup for concurrent imaging of eight adult zebrafish

**Figure 1:**
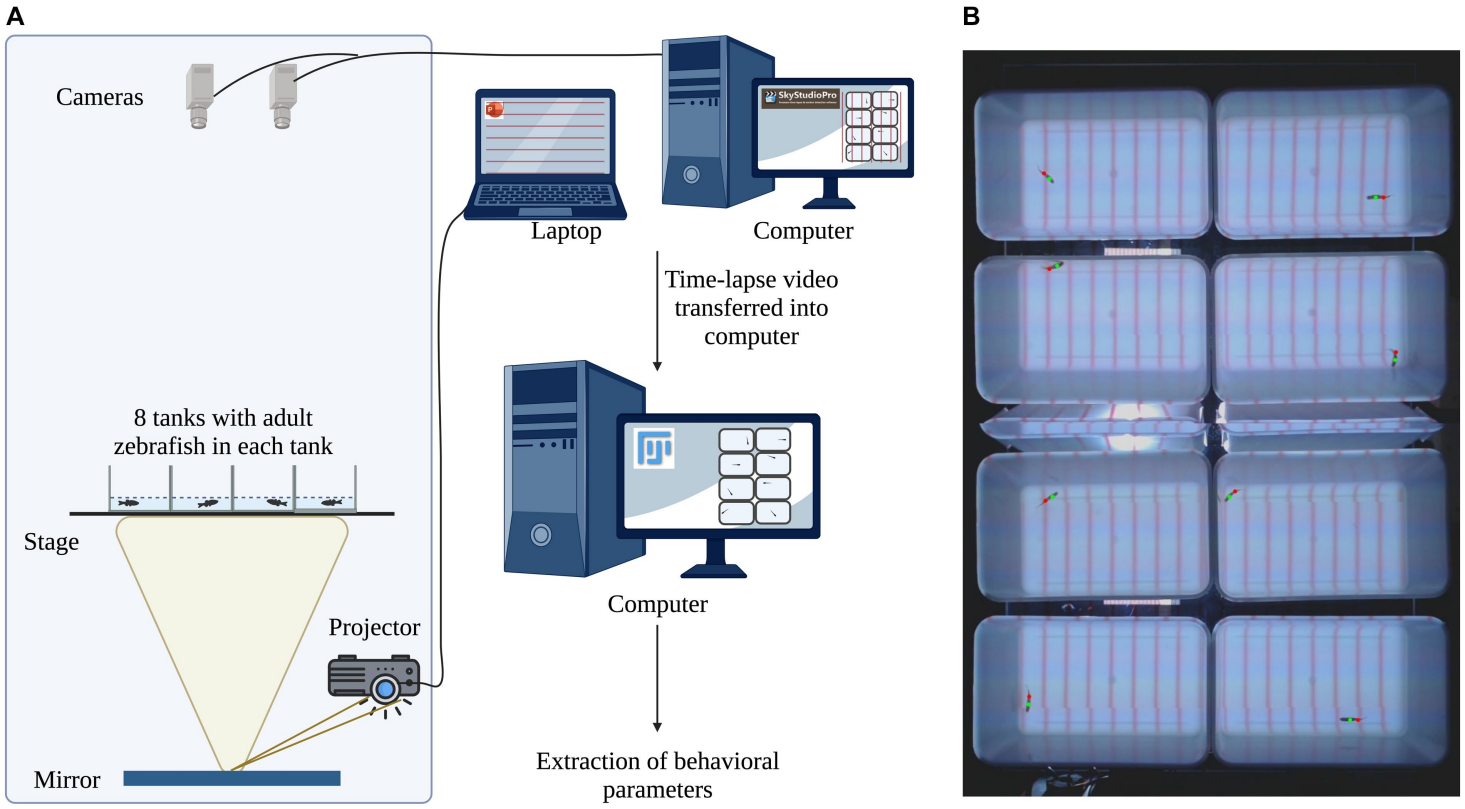
Imaging setup: **(A)** Behavioral imaging setup: the imaging setup consists of a projector attached to a laptop, a mirror, a stage, and two overhead web cameras that record zebrafish locations during the experiment. The projection of visual stimuli is achieved by the projector that is connected to a laptop computer with PowerPoint software. The projector illuminates the bottom of the tank by reflecting off the surface of the mirror, which lays flat on the ground. The two web cameras are connected to a computer and the SkyStudioPro application is used to control the camera feed from both the web cameras. The imaging data are transferred to another computer and analyzed post-imaging using a macro developed in Fiji/ImageJ. **(B)** Detection of adult zebrafish: Using a Fiji/ImageJ macro, adult zebrafish were detected. In the example of a representative acquired image, the centroid (red dot) and the center of bounding boxes (green dot) are shown for each fish/tank. Created with BioRender.com.

In the current study, we developed an imaging system for the behavioral analysis of adult zebrafish. This imaging system for behavioral analysis of adult zebrafish can hold eight tanks and image them concurrently. A wooden cabinet (Height = 64 in, Width = 39 in, Depth = 33 in) is used to hold the components of the imaging system. The wooden cabinet helps to maintain adult zebrafish in optimum environmental conditions and reduces external artifacts such as changes in ambient light, etc. (Gore et al., 2023). The imaging system (Figure 1A) consists of two web cameras (Logitech C922x Pro) mounted on the ceiling of the box, a transparent acrylic stage (Amazon, B00C13Z874) for holding tanks, a mirror, and a M5 LED Pico projector (Aaxa Technologies). The holding tanks are made of white opaque polypropylene material (Thomas Scientific Cat No. 1113H35, L = 11.5 in, W = 7.5 in, and H = 5 in). The projector was used as an LED light source (900 lumens) for background illumination (brightness intensity of 350 Lux) and the projection of visual stimuli. The projector illuminates the bottom of the tanks via a mirror that lies flat on the floor. The projector is connected to a laptop computer with PowerPoint software. Visual stimuli are shown to the adult zebrafish as PowerPoint presentations (Supplementary material 1). The acrylic stage is transparent, and the holding tanks are made of a white-colored opaque material which ensures that the fish do not see the projector or other fish in neighboring tanks (Supplementary material 2). The two cameras record the details of zebrafish location and are connected to a computer [Dell 12th Gen Intel(R) Core (TM) i7-12700 2.10 Gz]. The use of PowerPoint to present different visual stimuli to the adult zebrafish allows one to use a variety of stimuli and gives the flexibility of changing the stimuli for each tank or in a specific portion of the tank if needed. The current imaging system is robust and effective in producing high-contrast images, which is critical for detecting adult zebrafish (dark object) against the tank (light background) (Figure 1B). The current imaging system is a powerful tool for behavioral analysis with relatively inexpensive equipment, which is easy to use, and pliable enough for stimuli presentation. The animals were transferred to the imaging room an hour before the experiment started and were transferred back to a separate tank in the fish room after the experiment.

### Behavioral assay

**Figure 2:**
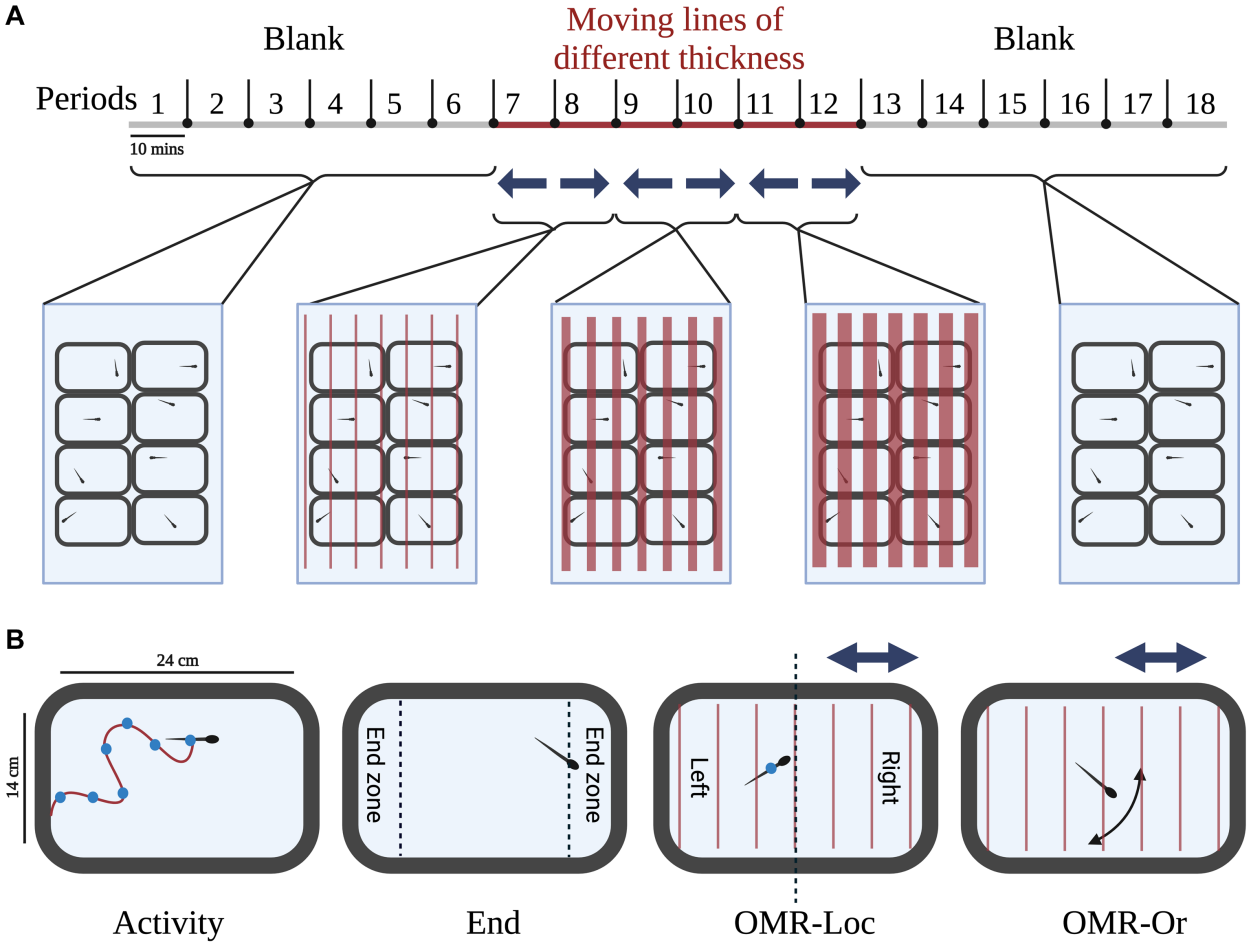
Experimental setup: **(A)** Timeline for the presentation of visual stimuli: the zebrafish adults were presented with a 3-h long PowerPoint presentation for studying behavioral response to visual stimuli. The 3-h long duration was broken down into 18 10-min periods for better dissection of behavior. During the first hour (Period 1–6), a blank slide was presented, to get baseline measures. Following the first hour, moving lines of different thicknesses and speed were presented during the second hour (Periods 7–12). During the odd periods (i.e., 7, 9, and 11) lines moved from right to left; while during the even periods (8, 10, and 12), the direction was reversed (left to right). Following the visual stimulus periods, the zebrafish adults were presented with a 1-h blank slide. **(B)** Behavioral measures: the coordinates of each zebrafish were used to calculate various behavioral parameters. Of particular interest were the measures of activity, end zone, Optomotor response location (OMR-Loc), and Optomotor response orientation (OMR-Or) described for each fish in the tank. Created with BioRender.com.

The behavioral assay used in the current study was used to understand the response of adult zebrafish to red-colored moving lines of different thicknesses. A 3-h long PowerPoint presentation (Supplementary material 1) was used to deliver visual stimuli to the adult zebrafish. Earlier studies in larvae show that they respond maximally to red-colored moving lines (fast-red) in comparison to blue or green-colored lines (Thorn et al., 2019; Edmister et al., 2022a,b; Gore et al., 2023). Therefore, we used red-colored lines of different thicknesses moving with the same speed in our PowerPoint presentation. The exact timeline of each stimulus is shown in Figure 2. The initial hour of the trial was considered the acclimatization phase during which zebrafish did not receive any visual stimuli (blank slide). The behaviors recorded during this period were used as a measure of baseline activity without any external stimuli. The 1-h blank slide was followed by 60 min of visual stimuli (moving lines of different thicknesses). Moving lines of different thicknesses and speed were presented during the second hour (Periods 7–12). In period 7, thin lines (1 mm thick lines set 7 mm apart, moving at the speed of 10.8 cm/s) moved from right to left. In period 8, thin lines moved toward the right. In period 9, medium-thickness lines (1 cm thick lines set 1 cm apart, moving at the speed of 13.8 cm/s) moved toward left and in period 10, medium-thickness lines moved toward right. In period 11, thick lines (2 cm thick lines set 2 cm apart, moving at the speed of 27.6 cm/s) moved from right to left. In period 12, thick lines moved from left to right. In summary, during the odd periods (i.e., 7, 9, and 11) lines moved from right to left; while during the even periods (8, 10, and 12), the direction was reversed (left to right). Following the visual stimulus periods, the zebrafish adults were presented with a 1-h blank slide. The sequence, timing, and details of the PowerPoint slide background are given in Supplementary material 1.

### Image acquisition

The image acquisition was similar to the method described in a previous study (Hernandez et al., 2023), which used similar setup to study mouse behavior. We used SkyStudioPro (version 1.1.0.30), a freely available program developed by DjSadhu in the Netherlands^1^ to control two webcams. SkyStudioPro combines the two camera feeds into a single recording. The optimal conditions used for image acquisition involved setting at a fixed white balance of 4,000, the focus at 0, and exposure at − 5. The capture size of each camera was set at 640 × 480 pixels and the size of the combined image was 640 × 960 pixels. The live and time-lapse codec was selected to ffdshow video codec. We acquired a timelapse video with the snapshot interval set at 1 s, movie speed at 30 fps, and autosave at 80,000 frames. With these settings, the 3-h long recording is saved as a ~ 200 MB AVI file and can be played back within 6 min at 30 × speed.

### Image analysis

An ImageJ/Fiji macro was developed for the automated analyses of the acquired AVI movies. ImageJ/Fiji are freely available online and the macro is included in Supplementary material 3. This macro can be installed in ImageJ or Fiji as a plugin (Open ImageJ or Fiji, Plugins, Install). The macro guides the user to open a movie as a virtual stack and collects information on the imaging files. It then uses this information to calculate the exact image interval. The macro then takes the first image in the stack, separates the color channels, and uses the red channel for subsequent analyses. In the red channel, the zebrafish are visible. Within each ROI, the macro applies a threshold and carries out a particle analysis, which filters out objects smaller and larger than zebrafish adults. The visual stimuli used in the current study are red-colored lines which do not show up in the red channel ensuring that only the zebrafish are detected as darker objects against lighter background. The particle analysis is set to measure various parameters of the zebrafish, including its centroid, bounding rectangle, and best-fitting ellipse. X and Y coordinates are logged in a “Results” file (Supplementary material 4). The macro repeats these steps for all images in the AVI file, generating a long list of X and Y coordinates. These coordinates are then used to calculate the following parameters: location of the animal, tank number, hour of imaging, and period of imaging (in 10-min increments), if a fish is located in its “End-zone” area, speed (in cm/s, using a 76 mm/70-pixel scaling factor), how often the fish swam more than 1 cm/s (% move), how often the right side of the fish is facing toward the center of the tank (RFC), the aspect ratio of the ellipse (major/minor axis). In total, the “Results” file has approximately 2.32 million data points (~80,000 rows × 29 columns).

### Data processing

Using MS Excel, we analyzed the 2.32 million data points of the ImageJ “Results” file into more informative tables and graphs. For the analysis, an ImageJ Results file (Supplementary material 4) is copied in the “Data” sheet of a MS Excel template (Supplementary material 5). This template contains calculations for six specific behavioral parameters—Speed (cm/s), Move (% time), End (time spent in 20% end on left and right side of the tank), RFC (% time), OMR-Loc (% time in the right half of the tank), and OMR-Or (% time oriented to the right) using the data (copied from “Results” file) tab. This template then averages specific behaviors per tank and per hour (e.g., speed in tank 8, hour 2) and then averages these behaviors in all tanks (the average speed of all zebrafish in the first hour). For each of the behavioral parameters, specific values are shown in graphs over time (18 10-min periods). A “Summary” table shows the following 24 behaviors in table format: (1) Speed = average speed during the first hour (cm/s) (2) M-1 h = movement during 1st hour, (3) M-Accl = measure of acclimation during the first hour, (4) M-Vis = average movement during the periods of visual stimuli, (5) M-St = Startle response to moving lines, (6) M-Hab = Movement during the presentation of moving lines during period 7 and 9, (7) M-Hab during period 9 and 11, (8) E-1 h = time spent in the end zones, (9) E-Accl = time spent in end zone during first and second 30-min of the first hour, (10) E-Vis = time spent in end zone during the presentation of moving lines, (11) E-St = Time spent in end zone when moving lines are presented for the first time, (12) E-Hab = Time spent in end zone during the presentation of moving lines (period 7 and 9), (13) E-Hab during period 9 and 11, (14) RFC = right side of the fish facing the center of the tank, (15) OMR-Loc = Optomotor location response during 7 and 8 periods, (16) OMR-Loc during periods 9 and 10, (17) OMR-Loc during periods 11 and 12, (18) OMR-Loc-Hab = OMR-Loc habituation during repeated exposure to lines moving in same direction (periods 7 and 9), (19) OMR-Loc-Hab during periods 9 and 11, (20) OMR-Or = Optomotor orientation response during 7 and 8 periods, (21) OMR-Or during periods 9 and 10, (22) OMR-Or during periods 11 and 12, (23) OMR-Or-Hab = OMR orientation habituation during repeated exposure to lines moving in same direction (periods 7 and 9), and (24) OMR-Or-Hab during periods 9 and 11.

### Statistical analysis

All the statistical tests and data visualization were conducted using Microsoft Excel 2016 and R statistical language. For each experiment, the normality and variance of the data were checked to assess if the assumptions of ANOVA were met. In cases where the data were non-normal and/or of unequal variance, a non-parametric Freidman’s rank sum and Wilcoxon signed-rank tests were used. To detect the significant effects of behavioral measures across different periods of the trial, we used repeated measures ANOVA with each behavioral measure as a dependent variable and periods/stimuli as a repeated measure. Following the ANOVA analysis, a *post-hoc* Bonferroni test was performed to identify significant effects in pairwise comparisons. All statistical tests were performed using (R v3.3.1, http://www.R-project.org) with supplementary installed packages “readr,” “ggplot2,” “ez,” “dplyr,” “tidyr,” and “ggpubr,” “rstatix,” and “tidyverse.”

## Results

### Adult zebrafish show unique behavioral profiles in response to visual stimuli

The behavioral response of adult zebrafish to different visual stimuli was recorded and summarized as a behavioral profile. A typical timeline for stimuli presentation for the current study is given in Figure 2A. Zebrafish adults were presented with a 3-h long PowerPoint—the first hour of no stimulus (blank slide), followed by an hour moving lines of different thicknesses in different directions and finally presented with an hour of no stimulus (blank slide). The four main behavioral parameters relevant in the current study were move (activity), end (time spent in the end zone), OMR-Loc which represents the location of adult zebrafish during the optomotor response, and OMR-Or which represents the orientation of adult zebrafish during the optomotor response (Figure 2B). Apart from these behavioral measures, we also quantified average speed and percent RFC (right side of the fish facing toward the center of the tank). Using the behavioral response of adult zebrafish across different stimuli presentations, we quantified 24 behavioral measures and built behavioral profiles for each animal. A typical behavioral profile for each wild-type adult zebrafish is given in Table 1. A positive move value was typically observed for adult zebrafish except for the move during startle response to visual stimuli (M-St p6-7). When first presented with the moving lines, adult zebrafish respond by increasing their activity during the initial 10-min period.

**Table 1:** Behavioral Profile (24 behaviors) of wild-type adult zebrafish.

**Exp Group**	**Speed (p1–6)**	**M-1h (p1–6)**	**M-Accl**	**M-Vis (p7–12)**	**M-St (p6–7)**	**M-Hab (p7–9)**	**M-Hab (p9–11)**	**E-1h (p1–6)**	**E-Accl**	**E-Vis (p7–12)**	**E-St (p6–7)**	**E-Hab (p7–9)**	**E-Hab (p9–11)**	CW (p1–6)	OMR-L (p7–8)	OMR-L (p9–10)	OMR-L (p11–12)	OMR-L-Hab (p7–9)	OMR-L-Hab (p9–11)	OMR-O (p7–8)	OMR-O (p9–10)	OMR-O (p11–12)	OMR-O-Hab (p7–9)	OMR-O-Hab (p9–11)
WT	3.327608132	55%	0%	60%	−5%	−7%	3%	62%	−1%	84%	−28%	15%	1%	54%	−96%	−30%	−64%	26%	0%	−24%	−12%	−22%	5%	−6%
WT	2.830924367	41%	−2%	59%	−19%	−5%	2%	67%	8%	79%	−30%	13%	9%	41%	−92%	−63%	−57%	11%	−2%	−37%	−34%	−25%	3%	8%
WT	2.48269152	30%	25%	51%	−53%	5%	23%	57%	−8%	77%	−32%	26%	0%	49%	−91%	26%	54%	42%	25%	−38%	−5%	5%	7%	5%
WT	2.207779949	23%	−2%	60%	−23%	−8%	−8%	60%	−10%	68%	−13%	12%	13%	49%	−83%	−75%	−10%	9%	−2%	−28%	−19%	−13%	5%	3%
WT	3.08552068	43%	3%	70%	−26%	−36%	6%	69%	15%	80%	5%	6%	5%	60%	−86%	−73%	−40%	6%	3%	−12%	−24%	−6%	−5%	8%
WT	2.81435937	41%	0%	70%	−55%	−9%	9%	77%	−5%	78%	−3%	33%	−3%	59%	−94%	−38%	−42%	55%	0%	−29%	−17%	−16%	12%	−2%
WT	1.437875412	14%	−1%	67%	−61%	−6%	14%	62%	−14%	69%	−25%	29%	8%	22%	−92%	−75%	−48%	10%	−1%	−51%	−23%	1%	18%	8%
WT	2.577432247	35%	11%	63%	−37%	6%	5%	61%	0%	65%	−3%	0%	−4%	49%	−29%	−18%	2%	13%	11%	−9%	−12%	−5%	−1%	7%
WT	2.440140508	26%	12%	25%	−10%	46%	−6%	44%	8%	79%	−37%	−16%	22%	50%	47%	−33%	−65%	−82%	28%	27%	−21%	−38%	−48%	15%
WT	2.625247097	38%	11%	29%	−50%	66%	−7%	68%	−10%	75%	−12%	−9%	−5%	47%	−44%	−18%	−57%	25%	−40%	−24%	−23%	−21%	−1%	0%
WT	2.892485765	34%	11%	26%	−27%	41%	−8%	58%	−13%	65%	−39%	48%	−4%	64%	−85%	−15%	−14%	67%	−13%	−51%	−16%	−4%	30%	8%
WT	3.295731215	55%	1%	34%	−62%	78%	−7%	49%	17%	62%	16%	3%	−12%	72%	−79%	−42%	−41%	30%	−5%	−9%	−16%	−17%	−7%	6%
WT	3.854794279	63%	4%	31%	−44%	66%	−11%	64%	3%	67%	−13%	27%	−4%	51%	−86%	−39%	−9%	28%	11%	−26%	−7%	−13%	12%	−2%
WT	3.569697566	55%	−3%	28%	−10%	58%	−6%	74%	−6%	76%	−15%	26%	−14%	52%	−84%	−8%	−26%	71%	−14%	−37%	−6%	3%	17%	9%
WT	5.812450811	70%	0%	34%	3%	60%	−8%	80%	−11%	73%	−16%	34%	−15%	52%	−73%	13%	−8%	68%	−17%	−34%	2%	−13%	28%	−12%
WT	3.4106223	53%	−9%	30%	−38%	72%	−8%	81%	5%	84%	8%	−13%	20%	52%	−85%	−33%	13%	52%	11%	−18%	2%	1%	14%	−17%
**Avg**	3.041585076	42%	4%	46%	−32%	27%	0%	65%	−1%	74%	−15%	15%	1%	51%	−72%	−33%	−26%	27%	0%	−25%	−14%	−11%	6%	2%
**N**	16	16	16	16	16	16	16	16	16	16	16	16	16	16	16	16	16	16	16	16	16	16	16	16
**SEM**	0.23585532	4%	2%	4%	5%	9%	2%	3%	2%	2%	4%	5%	3%	3%	9%	7%	8%	9%	4%	5%	2%	3%	5%	2%

Behavioral measures from multiple experiments were combined to generate a behavioral profile of WT adult zebrafish. Speed, Average speed during the first hour (cm/s); M-1h, Moved more than 3 cm/s during the first hour (% time); M-Accl, Compares movement in first and second 30 min as a measure of acclimation (p1–3 minus p4–6); M-Vis, Average movement in periods with visual stimuli (p7–12); M-St, Startle response to moving lines (p6–7 in % moved); M-Hab, Habituation to moving lines (p7–9 in % moved); M-Hab, Habituation to moving lines (p9–11 in % moved); E-1h, Located in the end zones during the first hour (% time in far left 20% or far right 20% of tank); E-Accl, Compares preference for end zones in first and second 30 min as a measure of acclimation (p1–3 minus p4–6); E-Vis, End zone location in periods with visual stimuli (p7–12); E-St, Startle response to moving lines (p6–7 in % end); E-Hab, Habituation to moving lines (p7–9 in % end); E-Hab, Habituation to moving lines (p9–11 in % end); RFC, Right side of the fish facing the center of the tank (p1–6); OMR_Loc, Optomotor response measured by changing Location in two subsequent 10-min periods; OMR_Loc-Hab, Habituation to moving lines (p7–9 and p9–11); OMR_Or, Optomotor response measured by changing orientation in two subsequent 10-min periods; OMR_Or-Hab, Habituation to moving lines (p7–9 and p9–11).

### Presentation of the visual stimuli elicits increased activity and time spent in the end zone

**Figure 3:**
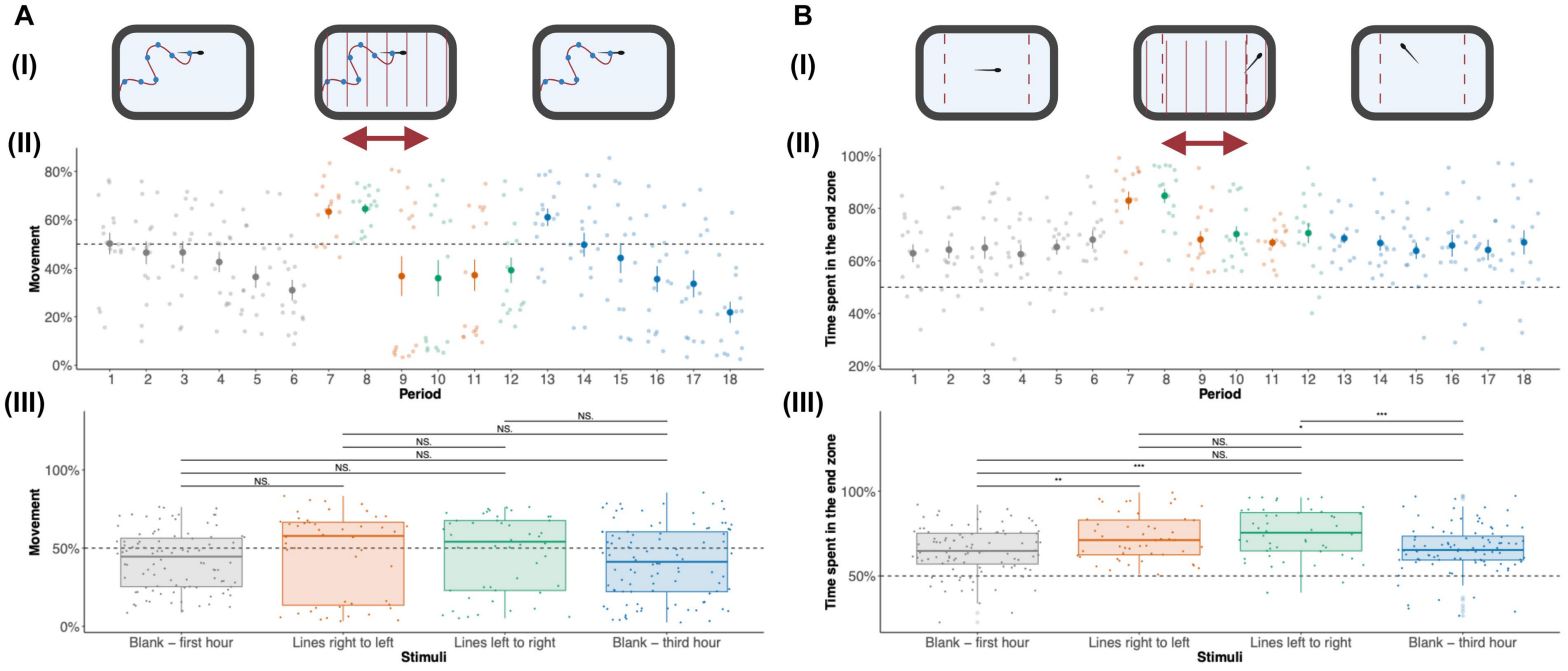
Effect of visual stimuli on activity and time spent in the end zone: **(A)** Activity of zebrafish adults: To study the movement trends of zebrafish adults in response to visual stimuli, we measured the amount of time spent that zebrafish adults spent moving. **(i)** The adult zebrafish activity was measured in the first-hour blank, visual stimuli (moving lines), and third-hour blank duration. **(ii)** The activity levels for 18 10-min periods are shown. The activity plot shows the average [± standard error (SE)] amount of time spent moving by adult zebrafish during the first hour of no stimuli (shown in gray), moving lines right to left (orange), moving lines left to right (green), and third hour of no stimuli (blue) for each of 18 10-min periods. The amount of movement is combined for each of the stimuli—blank first hour (gray), moving lines right to left (orange), moving lines left to right (green), and third hour of no stimuli (blue) in panel **(iii)**. The activity levels are shown as median ± Interquartile range (IQR). Blank (first hour) and Blank (third hour) were each an hour long while moving lines (left to right and right to left) were each 30 min long. Therefore, there are 96 data points (*n* = 16 × 6 periods) for each blank (first and third hour) stimuli and 48 data points (*n* = 16 × 3 periods) for moving lines (left to right and right to left) stimuli. The activity levels show statistical significance (*p* = 4.69 × 10^−10^) across periods but not across different stimuli. All the pairwise comparisons for periods are listed in Supplementary material 6. **(B)** Time spent in the end zone: the amount of time spent in the end zones was studied by calculating the amount of time spent in the left and right extremes (20% left and right). **(i)** The amount of time spent in the end zone by adult zebrafish was measured in the first-hour blank, moving lines, and third-hour blank duration. **(ii)** The time spent in the end zone for 18 10-min periods is shown. Time spent in the end zone [± standard error (SE)] by adult zebrafish during each period is shown. **(iii)** The amount of time spent in the end zone is combined for each of the stimuli—Blank first hour, moving lines right to left, moving lines left to right, and third hour of no stimuli. Blank (first hour) and Blank (third hour) were each an hour long while moving lines (left to right and right to left) were each 30 min long. Therefore, there are 96 data points (*n* = 16 × 6 periods) for each blank (first and third hour) stimuli and 48 data points (*n* = 16 × 3 periods) for moving lines (left to right and right to left) stimuli. Repeated measures ANOVA show significance across periods (*p* = 7.7 × 10^−7^) and stimuli (*p* = 8.86 × 10^−4^). The values are shown as median ± Interquartile range (IQR). Differences were considered statistically significant for *p* values less than 0.05. ^*^p < 0.05, ^**^p < 0.01, ^***^p < 0.001, ^****^p < 0.0001, and NS., Not significant (*p* > 0.05).

We measured the amount of time adult zebrafish spent moving across different visual stimuli (Figure 3Ai). A detailed activity profile of adult zebrafish along 18 periods is given in Figure 3Aii. During the initial hour of no stimuli (blank slide), a baseline activity of an average of 45% was observed. During the first 10-min period, increased activity was seen which can be explained by the tendency of adult zebrafish to explore novel environments. The activity gradually decreased along the subsequent periods of no stimulus. A sharp increase (around 65%) in activity is observed during periods (6 and 7) when the adult zebrafish receive thin moving lines in different directions. For the following subsequent periods of visual stimuli with moving lines (periods 9–12), the activity levels returned to baseline. In general, huge variation in activity levels was observed for periods 9–12. The decreased activity response to moving lines can be due to desensitization to visual stimuli. When presented with a blank slide following the moving lines, an increase in activity levels was observed (in period 13). The activity levels during the subsequent periods of the last hour showed a decline in activity levels. We also quantified the total activity levels during the first hour (blank—periods 1–6), moving lines (right to left, i.e., for periods 7, 9, and 11), moving lines (left to right, i.e., for periods 8, 10, and 12), and last hour (blank—periods 13–18) represented in Figure 3Aiii. Generally, increased activity levels were observed for moving lines stimuli presentation in comparison to the blank slide. We ran repeated measures ANOVA to check the effect of activity across different periods and stimuli. A significant effect (*p* = 4.69 × 10^−10^) was observed across periods but not across different stimuli. A Bonferroni *post-hoc* test showed significant differences in activity levels across different periods (Supplementary material 6).

Another important behavioral measure was the amount of time adult zebrafish spent in the end zone. We quantified the amount of time adult zebrafish spent in the end zone of the tank across different stimuli (Figure 3Bi). The amount of time spent by adult zebrafish in the end zone across 18 periods is shown in Figure 3Bii. During the initial hour of no stimuli, adult zebrafish spent 60% of their time in the end zone. When presented with moving lines during periods 6 and 7, a surge in the amount of time spent in the end zone was observed. For subsequent periods of moving lines, the time spent in the end zone returned to baseline levels. For the last hour with no stimuli, the time spent in the end zone continued to follow the baseline value.

The amount of time spent in the end zone across different stimuli—during the first hour (blank), moving lines (right to left), moving lines (left to right), and last hour (blank) is represented in Figure 3Biii. Repeated measures ANOVA with time spent in the end zone as the dependent variable and periods as repeated measures revealed significant (*p* = 7.7 × 10^–7^) effects. *Post-hoc* test with Bonferroni adjustment for multiple comparisons following repeated measures ANOVA showed significant differences in end zone times across different periods (Supplementary material 6). We observed a significant (*p* = 8.86 × 10^−4^) effect of stimuli on end zone time. *Post-hoc* tests revealed significant effects for all combinations of moving lines in comparison to no stimuli (blank—first hour and blank—third hour). These results clearly indicate that moving lines result in the placement of zebrafish toward to end zone of the tank; an effect perhaps attributed to the optomotor response.

### Adult zebrafish show a robust optomotor response when presented with moving lines

**Figure 4:**
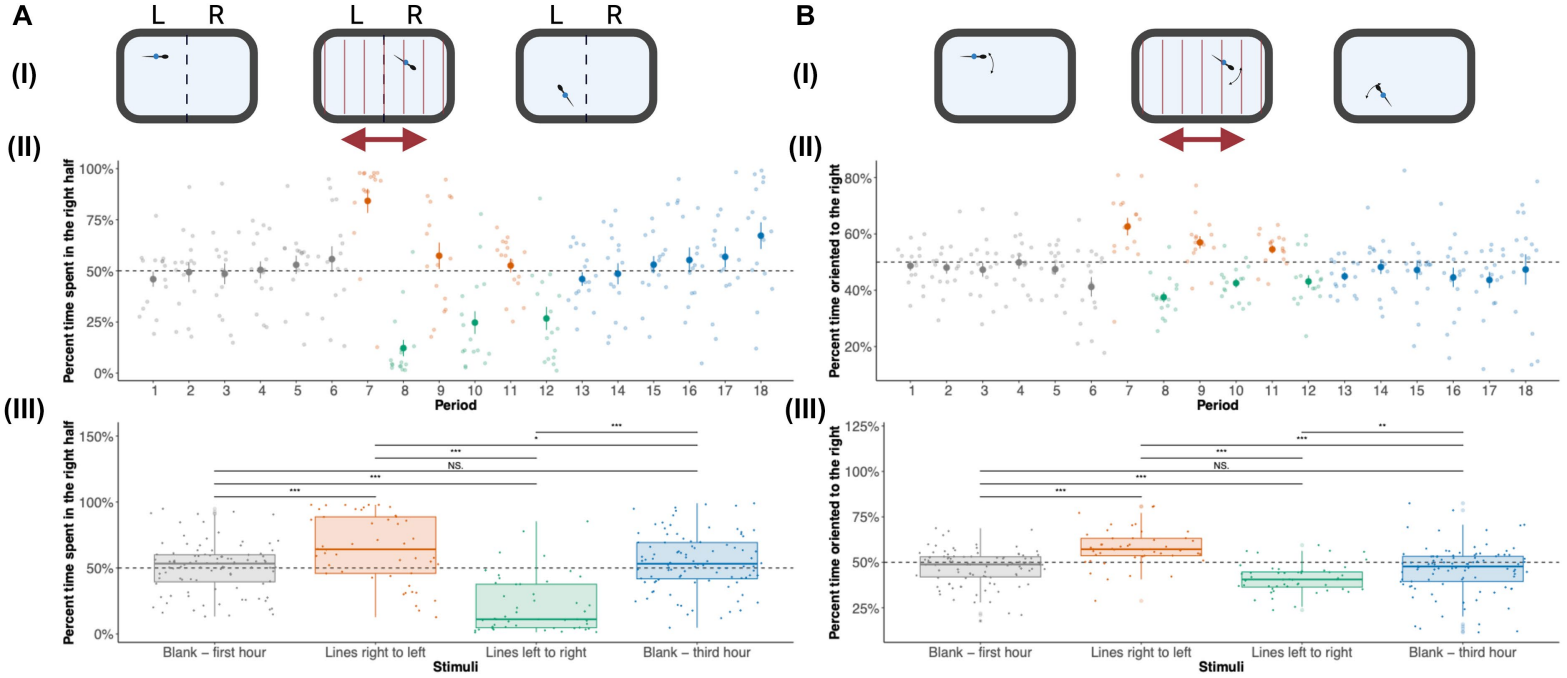
Effect of visual stimuli on Optomotor response (OMR): **(A)** OMR-Loc: **(i)** Zebrafish locations were used to calculate the amount of time spent on the right and left side of the tank during the first-hour blank, moving lines, and third-hour blank duration. **(ii)** The amount of time spent in the right half of the tank for each of the 10-min periods is shown. The OMR-Loc plot shows the average [± standard error (SE)] amount of time spent on the right side by adult zebrafish during the first hour of no stimuli, moving lines right to left, moving lines left to right, and third hour of no stimuli for each of 18 10-min periods. The time spent on the right side is combined for each of the stimuli—Blank first hour, moving lines right to left, moving lines left to right, and third hour of no stimuli in panel **(iii)**. These values are shown as median ± Interquartile range (IQR). Blank (first hour) and Blank (third hour) were each an hour long while moving lines (left to right and right to left) were each 30 min long. Therefore, there are 96 data points (*n* = 16 × 6 periods) for each blank (first and third hour) stimuli and 48 data points (*n* = 16 × 3 periods) for moving lines (left to right and right to left) stimuli. OMR-Loc was statistically significant across periods (*p* = 1.57 × 10^−23^) and stimuli (*p* = 4.32 × 10^−12^). The *p* values for all the pairwise comparisons are listed in Supplementary material 6. **(B)** OMR Orientation (OMR-Or): **(i)** The orientation of zebrafish adults was used to calculate the amount of time zebrafish are oriented toward the right and left during the first-hour blank, moving lines, and third-hour blank duration. **(ii)** The percent time oriented to the right for 18 10-min periods is shown. The OMR-Or plot shows the average [± standard error (SE)] amount of time spent oriented toward the right by adult zebrafish during the first hour of no stimuli, moving lines right to left, moving lines left to right, and third hour of no stimuli for each of 18 10-min periods. **(iii)** The percent time spent oriented to the right is combined for each of the stimuli—Blank first hour, moving lines right to left, moving lines left to right, and third hour of no stimuli. Blank (first hour) and Blank (third hour) were each an hour long while moving lines (left to right and right to left) were each 30 min long. Therefore, there are 96 data points (*n* = 16 × 6 periods) for each blank (first and third hour) stimuli and 48 data points (*n* = 16 × 3 periods) for moving lines (left to right and right to left) stimuli. These values are shown as median ± Interquartile range (IQR). OMR-Or was statistically significant across periods (*p* = 2.16 × 10^−10^) and stimuli (*p* = 6.2 × 10^−8^). Differences were considered statistically significant for *p* values less than 0.05. ^*^p < 0.05, ^**^p < 0.01, ^***^p < 0.001, ^****^p < 0.0001; NS, Not significant (*p* > 0.05).

Zebrafish larvae (5–7 dpf) are known to show a robust positive optomotor response where they swim in the direction of moving lines. In adult zebrafish, we observe a robust negative optomotor response. Adult zebrafish were imaged without visual stimuli (blank slide) and moving lines of different thicknesses. In order to quantify their optomotor response, we measured their location (Figure 4Ai) and orientation (Figure 4Bi) values during the presentation of visual stimuli. Figure 4Aii shows the location of zebrafish larvae across periods 1–18. During the first hour (periods 1–6) without stimuli, adult zebrafish spent an equal amount of time (50%) in the right and left half of the tank. When adult zebrafish are presented with lines that move toward the left (periods 7, 9, and 11), they swim in the opposite direction, toward the right side of the tank. During periods 8, 10, and 12, adult zebrafish receive moving lines toward the right, and they swim toward the left side of the tank, maintaining the negative optomotor response. During period 6, when adult zebrafish received moving lines for the first time, a large increase (approximately 85%) in the time spent in the right half of the tank was observed. When the direction of the moving lines is switched, there is an immediate switch in preference for the left side of the tank (approximately 15% of time spent on the right side). This pattern of a negative optomotor response is persistent for repeated bouts of moving lines of different thicknesses suggesting that repeated exposure to moving lines desensitizes the OMR response. The amount of time spent in the right half returns to baseline levels (around 50%) during the last hour without visual stimuli. When the OMR-Loc was combined for each stimulus, a clear preference for the right side was observed when moving lines were presented from right to left, and a switch in preference to the left side was seen for a switch in the direction of moving lines (Figure 4Aiii). For the first and third hours, without visual stimuli, adult zebrafish spent 50% of the time on the right side of the tank. Repeated measures ANOVA showed a significant effect of periods on the OMR-Loc (*p* = 1.57 × 10^−23^). *Post-hoc* analysis with Bonferroni adjustment showed a significant effect of OMR-Loc across different periods (Supplementary material 6). Repeated measures ANOVA with OMR Loc as the dependent variable and Stimuli as the repeated measures showed significant effects (*p* = 4.32 × 10^−12^). Pairwise multiple comparisons showed significant differences across all the combinations except for the blank first and blank third hour.

Optomotor responses can be measured by location as well as orientation. Measurements of orientation can reveal if the fish are oriented in the same direction as the moving lines (positive OMR) or in the opposite direction as the moving lines (negative OMR). Figure 4Bii shows the orientation of zebrafish larvae across periods 1–18. The pattern observed for the orientation of adult zebrafish across periods was similar to the OMR response seen for location. In the first hour (periods 1–6) when adult zebrafish received no visual stimuli, they spent 50% of the time oriented toward the right. When adult zebrafish receive moving lines from right to left (periods 7, 9, and 11), they orient themselves toward the right. For periods 8, 10, and 12 when adult zebrafish received moving lines from left to right, they oriented toward the left thus maintaining the negative optomotor response. During the third hour, i.e., for periods 13–18, adult zebrafish orient 50% toward the right. There is a reduction in the OMR response when presented with repeated visual stimuli. For each stimulus, a strong orientation response toward the right was observed when moving lines were presented from right to left, and a switch in orientation to the left was seen when the direction of moving lines was switched (Figure 4Biii). When no stimuli were presented (first and third hours), adult zebrafish spent 50% of their time oriented toward the right. Repeated measures ANOVA showed a significant effect of periods on the OMR orientation (*p* = 2.16 × 10^−10^). *Post-hoc* analysis with Bonferroni adjustment showed a significant effect of OMR-Loc across different periods (Supplementary material 6). Repeated measures ANOVA with OMR Orientation as the dependent variable and Stimuli as the repeated measures showed significant effects (*p* = 6.2 × 10^−8^). Pairwise multiple comparisons showed significant differences across all the combinations except for the blank first and blank third hour.

### Zebrafish preferably spend more time along the walls of the tank

**Figure 5:**
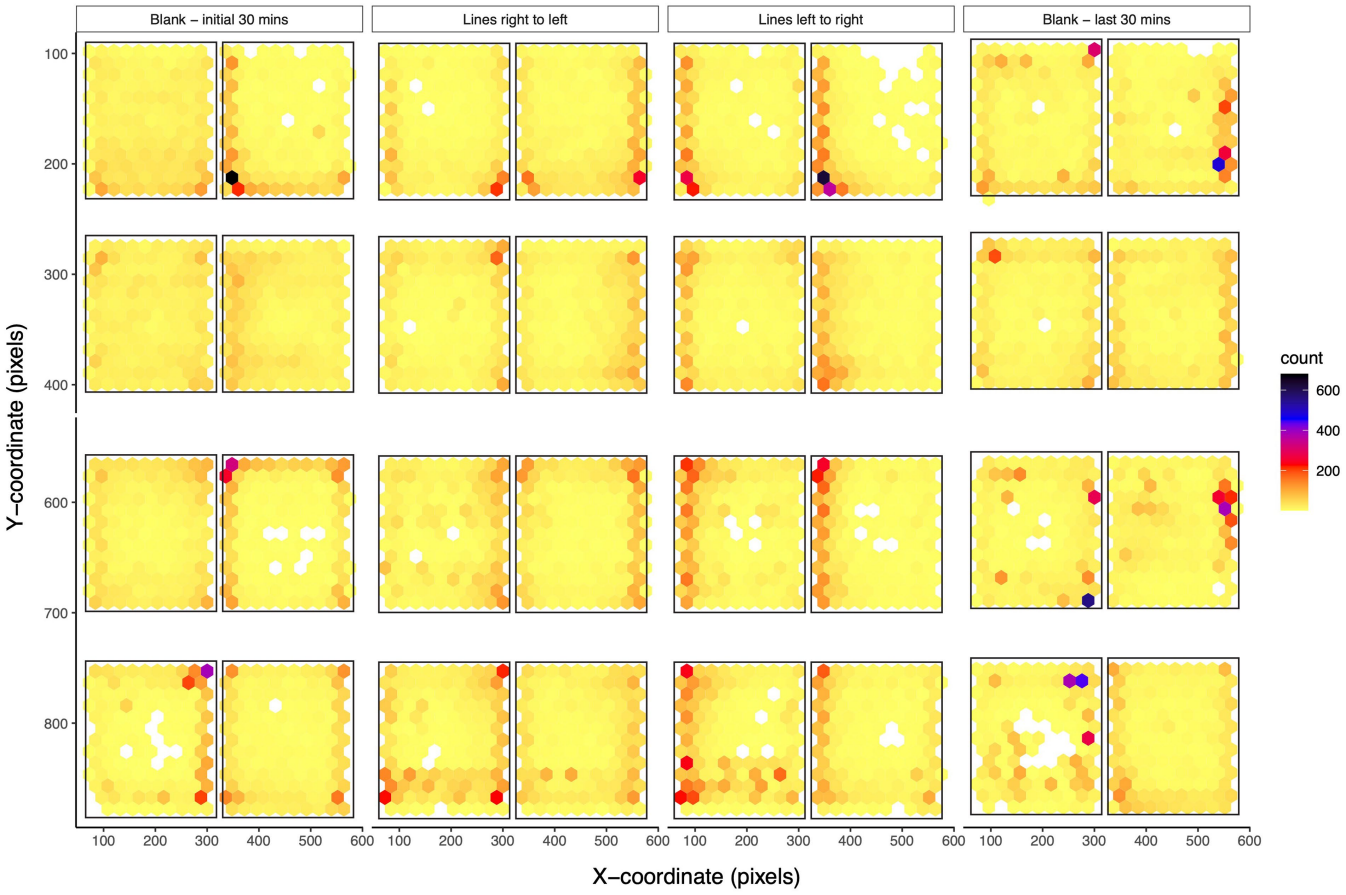
Spatial characteristics of adult zebrafish: The density plot for coordinates of adult zebrafish in eight-tank fashion are shown for the first 30 min of no stimuli, moving lines right to left, moving lines left to right, and the last 30 min of no stimuli. The plot divides the plane into regular hexagons and counts the number of cases in each hexagon. During the initial acclimation phase, zebrafish adults swam all over the space of the tank and explored the tank. When presented with moving lines, zebrafish were located more toward the end zones (left/right edge of the tank). During the presentation of moving lines, adult zebrafish are in the region from where the lines start moving. During the last 30 min of the trial, their swimming preference returned to the baseline levels.

Zebrafish adults show complex behaviors, and their space utilization gives valuable insights about their behaviors. We looked at the spatial characteristics of the adult zebrafish during different visual stimuli and counted the number of times zebrafish were located at a particular location in the tank (Figure 5). Generally, zebrafish adults preferred to stay around the edges of the tanks rather than toward the center. During the initial 30 min, zebrafish adults swam all over the space of the tank but were located more toward the walls of the tank suggesting initial exploration of the novel environment. When zebrafish received moving lines, their space utilization characteristics changed, and they were located more toward the end zones (left/right edge of the tank) as seen by an increase in the counts along the ends (Figure 5). Moving lines from right to left resulted in the placement of zebrafish toward the right end while the opposite direction of moving lines resulted in a movement toward the left end. Zebrafish returned to their exploratory swimming preference during the last 30 min of no stimuli. Spatial characteristics confirm the observed effects of various behavioral measurements across various stimuli.

## Discussion

Automated measurements and accurate quantification are critical in studying the processes involved in behavior (Krakauer et al., 2017). Zebrafish are an excellent model for genetic and molecular studies (Choe and Kim, 2023) and their application for studying behavior is sparse (Gerlai, 2023). With the availability of advanced imaging and computational tools, one can resolve complex behaviors into simpler bits, which was not possible in the past. The quantitative analysis of behavior provides a great way to not only study animal behavior in the natural environment but also to check the response of animals to various stimuli by dissecting a complex set of behaviors into individual well-defined easy to easy-to-interpret components (Gore et al., 2023; Hernandez et al., 2023). The current study uses a robust, inexpensive, and flexible method for behavioral quantification of responses to visual stimuli in a relatively high-throughput format. We used red-colored moving lines of different thicknesses in different directions as visual stimuli. Even with a relatively simple experimental setup, we were able to extract 24 behavioral measures for building a behavioral profile that was unique to the wild-type animals. The current study focused on understanding in detail the effects of visual stimuli on selected behavioral measures—activity, end zone, OMR-Loc, and OMR orientation, in 10-min intervals across the experiment. The imaging method used in the current study shows how relatively simple stimuli can be used to extract a variety of behavioral measures and help us understand complex behavior in simpler components. Our behavioral system is relatively simple, minimalistic, and flexible enough making them well-suited for designing independent research projects for the undergraduates and graduate students in the laboratory. The ImageJ macro developed in the current study is available in the Supplementary material and can be modified further to accommodate multiple behaviors and demonstrates how one can study complex behaviors.

One of the important aspects of behavior involves understanding how animals respond to new and changing environments. The current study looked at the baseline activity levels across an hour of acclimation phase before a visual stimulus was presented. An hour of blank slide, without visual stimuli, let us look at the exploration and space utilization characteristics shown by the wild-type animals. Our results indicate that zebrafish when introduced in the experimental arena, spent more time exploring the environment as seen by increased activity and increased time spent along the edges of the wall. These results are consistent with other studies that used open-field tests to study locomotor effects in zebrafish (Stewart et al., 2012; Ahmad and Richardson, 2013). When we compared the activity levels during the 10-min periods of the first hour, the amount of time spent exploring decreased across the subsequent periods of the first hour suggesting that these animals habituate to the environment eventually (Stewart et al., 2013). When animals start receiving thin moving lines for the first time, they show increased activity that stays high for two consecutive periods (periods 6 and 7). It is the transition from blank to moving lines that elicits this increase in activity levels. In another set of experiments (data not shown), we changed the sequence of thicknesses of lines (from thick lines to thin lines), and we observed this sharp increase in activity levels to the first visual stimuli (thick moving lines) received by the adult zebrafish. This increase in activity after the presentation of moving lines suggests that the animals can adapt their behavior in response to visual cues and that these behavioral adaptations may be because of the complex nature of visual-motor feedback systems. We observe that animals respond to the transition from moving lines to the last hour of no stimuli by increasing their exploration. However, when we compare the activity levels during the 10-min intervals on the last hour, their activity decreases over time.

One of the striking findings observed in this study was a negative OMR to the presentation of moving lines with respect to the location and orientation of zebrafish adults. Previous studies from our lab used zebrafish larvae for the behavioral analysis and larvae consistently show a positive OMR (Richendrfer and Creton, 2013; Thorn et al., 2019; Edmister and Creton, 2022; Edmister et al., 2022a,b; Gore et al., 2023) i.e., they orient and swim in the direction of moving lines. Adult WT animals used in the current study showed a robust negative OMR with respect to location and orientation. This switch in OMR response observed in our studies is consistent with the findings of a study by Bak-Coleman et al., who found a positive OMR in 9–11 dpf larvae, a negative OMR in 16–18 dpf larvae, and a negative OMR in adult zebrafish (Bak-Coleman et al., 2015). The OMR is a reflexive behavior observed in many animal species and can be interpreted as an evolutionarily conserved mechanism for stabilizing themselves with respect to the visual environment. The switch in OMR reversal observed in adult zebrafish can be difficult to interpret, however, one explanation is that this is perhaps a result of increased exploration of the environment during development. It is interesting that OMR switches during the development of zebrafish larvae, between 11 and 16 dpf, and opens new avenues for studying mechanisms underlying OMR behavior in development. In contrast to the findings of the current study, other studies show that adult zebrafish show a positive OMR (LeFauve et al., 2021), and further studies are needed to better understand these opposing findings in adult zebrafish We also found out that adult zebrafish show reduced OMR across visual stimuli. The moving lines in subsequent periods were of different thicknesses and the reduction of OMR can be due to differences in response to different thicknesses. However, results from another experiment (data not shown) used reverse order of presentation of moving lines (i.e., from thick lines to thin lines) and showed that zebrafish adults show robust negative OMR, and that the response reduces upon repeated presentation of moving lines suggesting that the reduction of OMR upon repeated presentation of moving lines is because of habituation to OMR. The reduced OMR across visual stimuli could be a habituation response to the moving lines; however, since the features of stimuli (speed and thickness of lines) changed across repeated exposures, the reduction in response can be because of the stimulus itself. While the scope of the current study was to understand the behavioral effects of visual stimuli in adult zebrafish, the use of males and females in the study allowed us to dissect behavior in a sex-specific fashion. The behavioral profile of males and females for 24 behavioral parameters looked similar but individual differences were only apparent when behaviors across different periods were considered. An interesting observation was that there were statistically significant sex-specific differences in the amount of time that animals spent in the end zone and OMR orientation parameters (Supplementary material 7). Particularly, these differences were pronounced during the first hour of the experiment; suggesting inherent differences in the way the environment is explored by each sex. However, more sex-specific studies in adult zebrafish are needed to check if this effect is observed consistently.

The high-throughput behavioral imaging system developed in the current study shows the importance of quantifying simple behaviors and opens opportunities to pinpoint the neural processes important in behavior. The finding of the current study builds on the existing literature available on behavioral effects observed in zebrafish adults. Understanding the baseline behavioral trends and patterns is critical to pinpoint the neural processes underlying behavior. Optomotor and other visually guided behaviors are well-studied in zebrafish larvae. Various features of OMR and downstream neural processes in zebrafish larvae have similarities with higher-order vertebrates (Naumann et al., 2016). With advances in whole brain studies in zebrafish larvae, it is evident that multiple levels of analyses from synapses to neuronal connectivity drives behaviors (Loring et al., 2020). In zebrafish, the retina extracts multiple features of the visual stimuli, and the integration and refinement of visual cues occur in the pretectum and other central brain areas (Yildizoglu et al., 2020). Visually guided behaviors are performed by many animals and the neural mechanism responsible for these behaviors seems to be evolutionarily conserved (Nakayama, 1985). Studying neural mechanisms responsible for simple behavior can help one understand similarities and differences between neural circuits across species and how neural circuits evolve in response to specific behaviors (Katz, 2011). Zebrafish larvae are used extensively for high-throughput drug screens (MacRae and Peterson, 2015) and extending the effects of selected drugs to adult zebrafish animals will help one to understand how these drugs act as animals age to provide valuable insights on the effects of these drugs across development. Furthermore, knowledge about the baseline behavioral measures will help us compare the behaviors of adult zebrafish in the diseased state models to dissect the mechanisms involved in the pathology of various neurological disorders. Due to the availability of genetic and molecular methods in combination with advanced behavioral analysis and computational tools, the use of zebrafish models in neuroscience research is expanding rapidly (Kalueff et al., 2013, 2014). Even though there are differences in behavioral responses observed in humans in comparison to zebrafish, various complex CNS traits are evolutionarily conserved suggesting that zebrafish may share common genetic and physiological factors with humans (Gerlai, 2021). With the automated behavioral analysis along with sophisticated computation tools available, zebrafish offers exciting opportunities to study biological mechanisms underlying behavior.

## Data availability statement

The original contributions presented in the study are included in the article/Supplementary material, further inquiries can be directed to the corresponding author.

## Ethics statement

The animal study was approved by Institutional Animal Care & Use Committee (IACUC), Brown University CARE. The study was conducted in accordance with the local legislation and institutional requirements.

## Author contributions

SG: Conceptualization, Data curation, Formal Analysis, Investigation, Methodology, Resources, Software, Validation, Visualization, Writing – original draft, Writing – review & editing. TR: Resources, Writing – review & editing. RC: Funding acquisition, Project administration, Supervision, Writing – review & editing, Investigation, Resources.

## Footnotes

^1^skystudiopro.com

## References

[ref1] Ahmad F. Richardson M. K. (2013). Exploratory behaviour in the open field test adapted for larval zebrafish: impact of environmental complexity. Behav. Process. 92, 88–98. doi: 10.1016/j.beproc.2012.10.014, PMID: 23123970

[ref2] Bak-Coleman J. Smith D. Coombs S. (2015). Going with, then against the flow: evidence against the optomotor hypothesis of fish rheotaxis. Anim. Behav. 107, 7–17. doi: 10.1016/j.anbehav.2015.06.007

[ref3] Bang P. I. Yelick P. C. Malicki J. J. Sewell W. F. (2002). High-throughput behavioral screening method for detecting auditory response defects in zebrafish. J. Neurosci. Methods 118, 177–187. doi: 10.1016/S0165-0270(02)00118-8, PMID: 12204308

[ref4] Barnard A. R. Nolan P. M. (2008). When clocks go bad: Neurobehavioural consequences of disrupted circadian timing. PLoS Genet. 4:e1000040. doi: 10.1371/journal.pgen.1000040, PMID: 18516223 PMC2295261

[ref5] Buske C. Gerlai R. (2011). Shoaling develops with age in zebrafish (*Danio rerio*). Prog. Neuro. Psychoph. 35, 1409–1415. doi: 10.1016/j.pnpbp.2010.09.003, PMID: 20837077 PMC3021101

[ref6] Choe S. K. Kim C. H. (2023). Zebrafish: a powerful model for genetics and genomics. Int. J. Mol. Sci. 24:8169. doi: 10.3390/ijms24098169, PMID: 37175875 PMC10179036

[ref7] Choi T. Y. Choi T. I. Lee Y. R. Choe S. K. Kim C. H. (2021). Zebrafish as an animal model for biomedical research. Exp. Mol. Med. 53, 310–317. doi: 10.1038/s12276-021-00571-5, PMID: 33649498 PMC8080808

[ref8] Clift D. Richendrfer H. Thorn R. J. Colwill R. M. Creton R. (2014). High-throughput analysis of behavior in zebrafish larvae: effects of feeding. Zebrafish 11, 455–461. doi: 10.1089/zeb.2014.0989, PMID: 25153037 PMC4172468

[ref9] DePasquale C. Franklin K. Jia Z. H. Jhaveri K. Buderman F. E. (2022). The effects of exploratory behavior on physical activity in a common animal model of human disease, zebrafish (*Danio rerio*). Front. Behav. Neurosci. 16:1020837. doi: 10.3389/fnbeh.2022.1020837, PMID: 36425283 PMC9679429

[ref10] DePasquale C. Kemerer N. White N. Yost M. Wolfkill J. Sturgill J.. (2021). The influence of an enriched environment in enhancing recognition memory in zebrafish (*Danio rerio*). Front. Vet. Sci. 8:749746. doi: 10.3389/fvets.2021.749746, PMID: 34869723 PMC8632956

[ref11] Easter J. S. S. Nicola G. N. (1996). The development of vision in the zebrafish (*Danio rerio*). Dev. Biol. 180, 646–663. doi: 10.1006/dbio.1996.0335 8954734

[ref12] Edmister S. T. Creton R. (2022). Modulation of calcineurin signaling during development. Dev. Neurobiol. 82, 505–516. doi: 10.1002/dneu.22895, PMID: 35785416 PMC9922048

[ref13] Edmister S. T. Ibrahim R. Kakodkar R. Kreiling J. A. Creton R. (2022a). A zebrafish model for calcineurin-dependent brain function. Behav. Brain Res. 416:113544. doi: 10.1016/j.bbr.2021.113544, PMID: 34425181 PMC8903086

[ref14] Edmister S. T. Del Rosario Hernández T. Ibrahim R. Brown C. A. Gore S. V. Kakodkar R.. (2022b). Novel use of FDA-approved drugs identified by cluster analysis of behavioral profiles. Sci. Rep. 12:6120. doi: 10.1038/s41598-022-10133-y, PMID: 35449173 PMC9023506

[ref15] Gerlai R. (2021). From genes to behavior: the question of evolutionary conservation and the role of ethology in the analysis of the zebrafish. Front. Neuroanat. 15:809967. doi: 10.3389/fnana.2021.809967, PMID: 34924966 PMC8675880

[ref16] Gerlai R. (2023). Zebrafish (*Danio rerio*): a newcomer with great promise in behavioral neuroscience. Neurosci. Biobehav. R. 144:104978. doi: 10.1016/j.neubiorev.2022.104978 36442644

[ref17] Gore S. V. Kakodkar R. Hernandez T. Edmister S. T. Creton R. (2023). Zebrafish larvae position tracker (Z-LaP tracker): a high-throughput deep-learning behavioral approach for the identification of calcineurin pathway-modulating drugs using zebrafish larvae. Sci. Rep. 13:3174. doi: 10.1038/s41598-023-30303-w, PMID: 36823315 PMC9950053

[ref18] Green J. Collins C. Kyzar E. J. Pham M. Roth A. Gaikwad S.. (2012). Automated high-throughput neurophenotyping of zebrafish social behavior. J. Neurosci. Methods 210, 266–271. doi: 10.1016/j.jneumeth.2012.07.017, PMID: 22884772

[ref19] Hernandez T. D. Joshi N. R. Gore S. V. Kreiling J. A. Creton R. (2023). An 8-cage imaging system for automated analyses of mouse behavior. Sci. Rep. 13:8113. doi: 10.1038/s41598-023-35322-1, PMID: 37208415 PMC10199054

[ref20] Hurd M. W. Debruyne J. Straume M. Cahill G. M. (1998). Circadian rhythms of locomotor activity in zebrafish. Physiol. Behav. 65, 465–472. doi: 10.1016/S0031-9384(98)00183-8 9877412

[ref21] Kalueff A. V. Echevarria D. J. Stewart A. M. (2014). Gaining translational momentum: more zebrafish models for neuroscience research preface. Prog. Neuro. Psychoph. 55, 1–6. doi: 10.1016/j.pnpbp.2014.01.022 24593944

[ref22] Kalueff A. V. Gebhardt M. Stewart A. M. Cachat J. M. Brimmer M. Chawla J. S.. (2013). Towards a comprehensive catalog of zebrafish behavior 1.0 and beyond. Zebrafish 10, 70–86. doi: 10.1089/zeb.2012.0861, PMID: 23590400 PMC3629777

[ref23] Kalueff A. V. Stewart A. M. Gerlai R. (2014). Zebrafish as an emerging model for studying complex brain disorders. Trends Pharmacol. Sci. 35, 63–75. doi: 10.1016/j.tips.2013.12.002, PMID: 24412421 PMC3913794

[ref24] Katz P. S. (2011). Neural mechanisms underlying the evolvability of behaviour. Philos. Trans. Roy. Soc. B. Biol. Sci. 366, 2086–2099. doi: 10.1098/rstb.2010.0336, PMID: 21690127 PMC3130364

[ref25] Kimmel C. B. Patterson J. Kimmel R. O. (1974). Development and behavioral characteristics of startle response in Zebra fish. Dev. Psychobiol. 7, 47–60. doi: 10.1002/dev.420070109, PMID: 4812270

[ref26] Krakauer J. W. Ghazanfar A. A. Gomez-Marin A. MacIver M. A. Poeppel D. (2017). Neuroscience needs behavior: correcting a reductionist Bias. Neuron 93, 480–490. doi: 10.1016/j.neuron.2016.12.041, PMID: 28182904

[ref27] LeFauve M. K. Rowe C. J. Crowley-Perry M. Wiegand J. L. Shapiro A. G. Connaughton V. P. (2021). Using a variant of the optomotor response as a visual defect detection assay in zebrafish. J. Biol. Methods 8:e144. doi: 10.14440/jbm.2021.341, PMID: 33604396 PMC7884848

[ref28] Loring M. D. Thomson E. E. Naumann E. A. (2020). Whole-brain interactions underlying zebrafish behavior. Curr. Opin. Neurobiol. 65, 88–99. doi: 10.1016/j.conb.2020.09.011, PMID: 33221591 PMC10697041

[ref29] MacRae C. A. Peterson R. T. (2015). Zebrafish as tools for drug discovery. Nat. Rev. Drug Discov. 14, 721–731. doi: 10.1038/nrd4627 26361349

[ref30] Maree L. Leonardis E. Gepshtein S. Albright T. Hitchcock K. Andrews N.. (2023). Quantifying behavior using deep learning. Biol. Psychiatry 93:S7. doi: 10.1016/j.biopsych.2023.02.038

[ref31] Mathis A. (2021). Deep learning tools for the analysis of movement, identity and behavior. Integr. Comp. Biol. 61, E581–E582.

[ref32] Mathis M. W. Mathis A. (2020). Deep learning tools for the measurement of animal behavior in neuroscience. Curr. Opin. Neurobiol. 60, 1–11. doi: 10.1016/j.conb.2019.10.008, PMID: 31791006

[ref33] Nakayama K. (1985). Biological image motion processing: a review. Vis. Res. 25, 625–660. doi: 10.1016/0042-6989(85)90171-3 3895725

[ref34] Nath T. Mathis A. Chen A. C. Patel A. Bethge M. Mathis M. W. (2019). Using DeepLabCut for 3D markerless pose estimation across species and behaviors. Nat. Protoc. 14, 2152–2176. doi: 10.1038/s41596-019-0176-0, PMID: 31227823

[ref35] Naumann E. A. Fitzgerald J. E. Dunn T. W. Rihel J. Sompolinsky H. Engert F. From whole-brain data to functional circuit models: the zebrafish optomotor response. Cell 167, 947–960. doi: 10.1016/j.cell.2016.10.019, PMID: 27814522 PMC5111816

[ref36] Neuhauss S. C. (2010) in Fish Physiology. eds. Perry S. F. Ekker M. Farrell A. P. Brauner C. J., vol. 29 (Academic Press), 81–122.

[ref37] Norton W. Bally-Cuif L. (2010). Adult zebrafish as a model organism for behavioural genetics. BMC Neurosci. 11, 1–11. doi: 10.1186/1471-2202-11-90, PMID: 20678210 PMC2919542

[ref38] Pereira T. D. Shaevitz J. W. Murthy M. (2020). Quantifying behavior to understand the brain. Nat. Neurosci. 23, 1537–1549. doi: 10.1038/s41593-020-00734-z, PMID: 33169033 PMC7780298

[ref39] Perez-Escudero A. Vicente-Page J. Hinz R. C. Arganda S. de Polavieja G. G. (2014). idTracker: tracking individuals in a group by automatic identification of unmarked animals. Nat. Methods 11, 743–748. doi: 10.1038/Nmeth.2994, PMID: 24880877

[ref40] Portugues R. Engert F. (2009). The neural basis of visual behaviors in the larval zebrafish. Curr. Opin. Neurobiol. 19, 644–647. doi: 10.1016/j.conb.2009.10.007, PMID: 19896836 PMC4524571

[ref41] Richendrfer H. Creton R. (2013). Automated high-throughput behavioral analyses in zebrafish larvae. J. Vis. Exp.:e50622. doi: 10.3791/50622, PMID: 23851916 PMC3731428

[ref42] Richendrfer H. Creton R. (2018). Cluster analysis profiling of behaviors in zebrafish larvae treated with antidepressants and pesticides. Neurotoxicol. Teratol. 69, 54–62. doi: 10.1016/j.ntt.2017.10.009, PMID: 29101052 PMC5930167

[ref43] Soussi-Yanicostas N. (2022). Zebrafish as a model for neurological disorders. Int. J. Mol. Sci. 23:8169. doi: 10.3390/ijms23084321, PMID: 35457137 PMC9025646

[ref44] Stewart A. M. Braubach O. Spitsbergen J. Gerlai R. Kalueffl A. V. (2014). Zebrafish models for translational neuroscience research: from tank to bedside. Trends Neurosci. 37, 264–278. doi: 10.1016/j.tins.2014.02.011, PMID: 24726051 PMC4039217

[ref45] Stewart A. M. Cachat J. Green J. Gaikwad S. Kyzar E. Roth A.. (2013). Constructing the habituome for phenotype-driven zebrafish research. Behav. Brain Res. 236, 110–117. doi: 10.1016/j.bbr.2012.08.026 22944516

[ref46] Stewart A. M. Gaikwad S. Kyzar E. Kalueff A. V. (2012). Understanding spatio-temporal strategies of adult zebrafish exploration in the open field test. Brain Res. 1451, 44–52. doi: 10.1016/j.brainres.2012.02.064, PMID: 22459042

[ref47] Stewart A. M. Gerlai R. Kalueff A. V. (2015). Developing highER-throughput zebrafish screens for in-vivo CNS drug discovery. Front. Behav. Neurosci. 9:14. doi: 10.3389/fnbeh.2015.00014, PMID: 25729356 PMC4325915

[ref48] Sykes D. J. Suriyampola P. S. Martins E. P. (2018). Recent experience impacts social behavior in a novel context by adult zebrafish (*Danio rerio*). PLoS One 13:e0204994. doi: 10.1371/journal.pone.0204994, PMID: 30335773 PMC6193632

[ref49] Thorn R. J. Dombroski A. Eller K. Dominguez-Gonzalez T. M. Clift D. E. Baek P.. (2019). Analysis of vertebrate vision in a 384-well imaging system. Sci. Rep. 9:13989. doi: 10.1038/s41598-019-50372-0, PMID: 31562366 PMC6764987

[ref50] Yildizoglu T. Riegler C. Fitzgerald J. E. Portugues R. (2020). A neural representation of naturalistic motion-guided behavior in the zebrafish brain. Curr. Biol. 30:e2326. doi: 10.1016/j.cub.2020.04.043, PMID: 32386533 PMC7314654

