# Supplementary material for: Behavioral effects of visual stimuli in adult zebrafish using a novel eight-tank imaging system
Source: Front Behav Neurosci. 2024 Mar 11;18:1320126. doi: 10.3389/fnbeh.2024.1320126 (PMC10962262; doi:10.3389/fnbeh.2024.1320126)
Supplement: Supplementary file 1 [file Data_Sheet_1.ZIP › Supplementary_data_1312024/Supp_file_1_Adult_ZF_Powerpoint.pptx]

## Slide 1
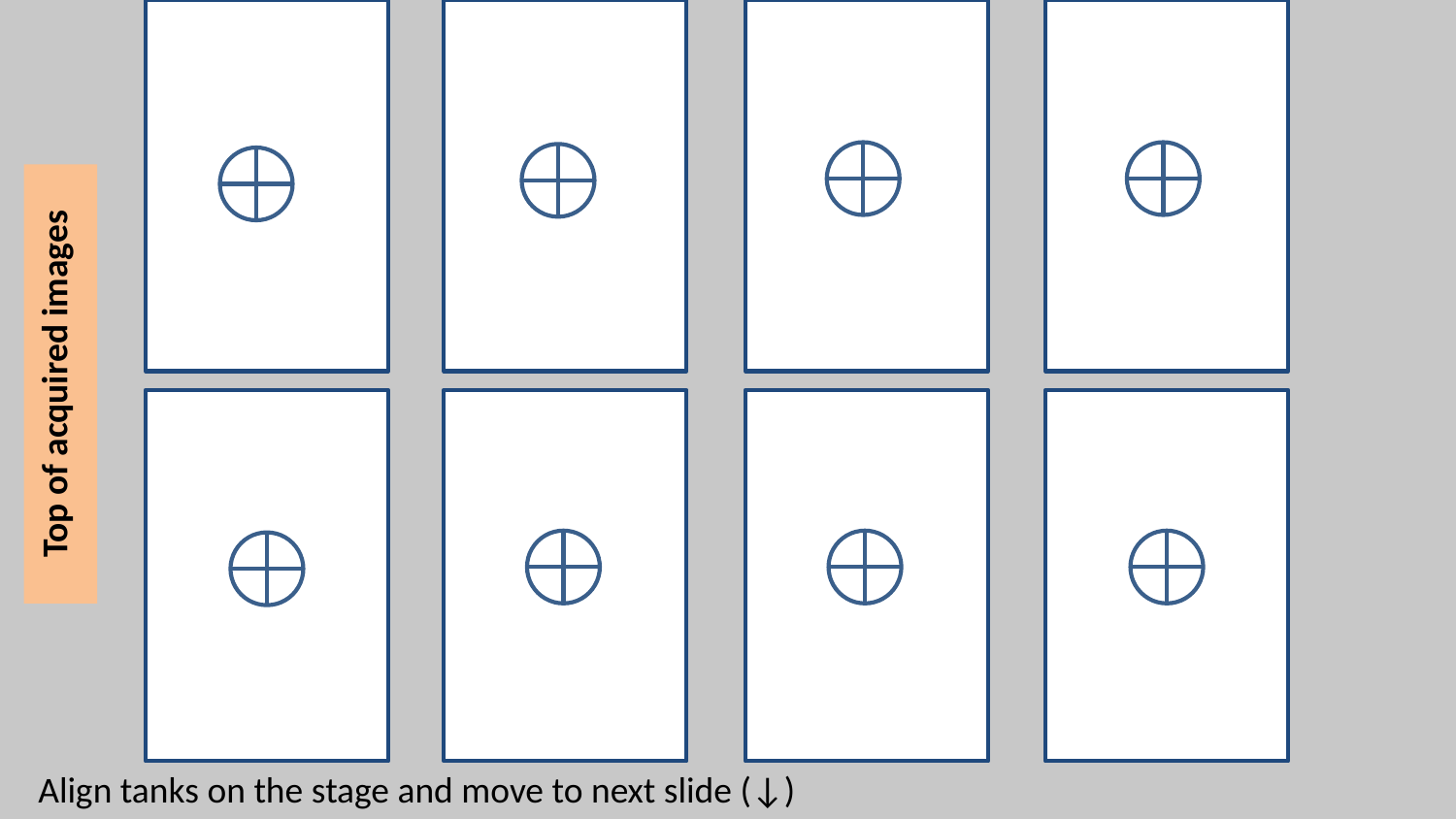

,,
Top of acquired images
Align tanks on the stage and move to next slide (↓)

## Slide 2
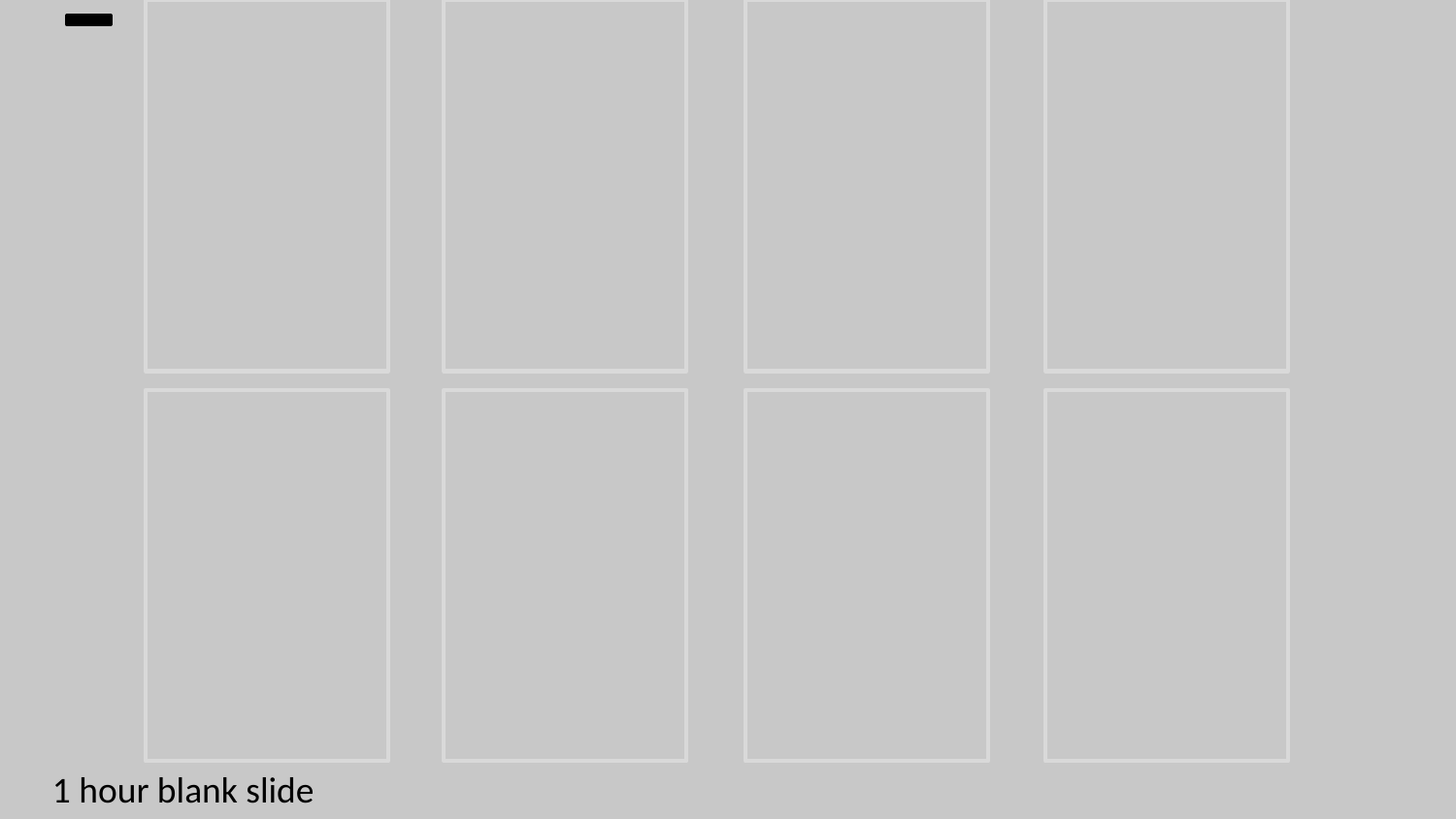

1 hour blank slide

## Slide 3
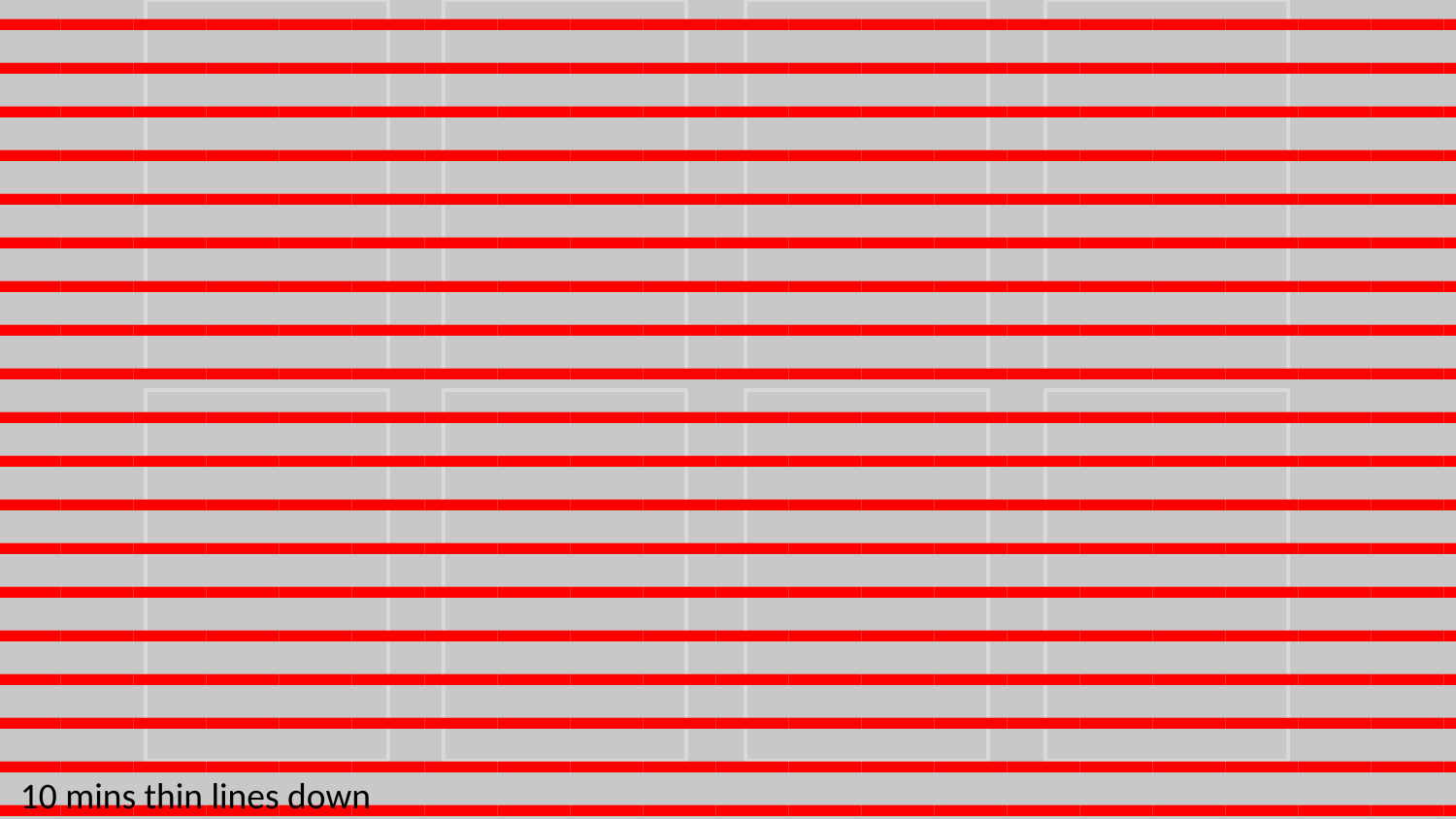

▬▬▬▬▬▬▬▬▬▬▬▬▬▬▬▬▬▬▬▬▬▬▬▬▬▬▬▬▬▬▬▬▬▬▬▬▬▬▬▬▬▬▬▬▬▬▬▬▬▬▬▬▬
▬▬▬▬▬▬▬▬▬▬▬▬▬▬▬▬▬▬▬▬▬▬▬▬▬▬▬▬▬▬▬▬▬▬▬▬▬▬▬▬▬▬▬▬▬▬▬▬▬▬▬▬▬
▬▬▬▬▬▬▬▬▬▬▬▬▬▬▬▬▬▬▬▬▬▬▬▬▬▬▬▬▬▬▬▬▬▬▬▬▬▬▬▬▬▬▬▬▬▬▬▬▬▬▬▬▬
▬▬▬▬▬▬▬▬▬▬▬▬▬▬▬▬▬▬▬▬▬▬▬▬▬▬▬▬▬▬▬▬▬▬▬▬▬▬▬▬▬▬▬▬▬▬▬▬▬▬▬▬▬
▬▬▬▬▬▬▬▬▬▬▬▬▬▬▬▬▬▬▬▬▬▬▬▬▬▬▬▬▬▬▬▬▬▬▬▬▬▬▬▬▬▬▬▬▬▬▬▬▬▬▬▬▬
▬▬▬▬▬▬▬▬▬▬▬▬▬▬▬▬▬▬▬▬▬▬▬▬▬▬▬▬▬▬▬▬▬▬▬▬▬▬▬▬▬▬▬▬▬▬▬▬▬▬▬▬▬
▬▬▬▬▬▬▬▬▬▬▬▬▬▬▬▬▬▬▬▬▬▬▬▬▬▬▬▬▬▬▬▬▬▬▬▬▬▬▬▬▬▬▬▬▬▬▬▬▬▬▬▬▬
▬▬▬▬▬▬▬▬▬▬▬▬▬▬▬▬▬▬▬▬▬▬▬▬▬▬▬▬▬▬▬▬▬▬▬▬▬▬▬▬▬▬▬▬▬▬▬▬▬▬▬▬▬
▬▬▬▬▬▬▬▬▬▬▬▬▬▬▬▬▬▬▬▬▬▬▬▬▬▬▬▬▬▬▬▬▬▬▬▬▬▬▬▬▬▬▬▬▬▬▬▬▬▬▬▬▬
▬▬▬▬▬▬▬▬▬▬▬▬▬▬▬▬▬▬▬▬▬▬▬▬▬▬▬▬▬▬▬▬▬▬▬▬▬▬▬▬▬▬▬▬▬▬▬▬▬▬▬▬▬
▬▬▬▬▬▬▬▬▬▬▬▬▬▬▬▬▬▬▬▬▬▬▬▬▬▬▬▬▬▬▬▬▬▬▬▬▬▬▬▬▬▬▬▬▬▬▬▬▬▬▬▬▬
▬▬▬▬▬▬▬▬▬▬▬▬▬▬▬▬▬▬▬▬▬▬▬▬▬▬▬▬▬▬▬▬▬▬▬▬▬▬▬▬▬▬▬▬▬▬▬▬▬▬▬▬▬
▬▬▬▬▬▬▬▬▬▬▬▬▬▬▬▬▬▬▬▬▬▬▬▬▬▬▬▬▬▬▬▬▬▬▬▬▬▬▬▬▬▬▬▬▬▬▬▬▬▬▬▬▬
▬▬▬▬▬▬▬▬▬▬▬▬▬▬▬▬▬▬▬▬▬▬▬▬▬▬▬▬▬▬▬▬▬▬▬▬▬▬▬▬▬▬▬▬▬▬▬▬▬▬▬▬▬
▬▬▬▬▬▬▬▬▬▬▬▬▬▬▬▬▬▬▬▬▬▬▬▬▬▬▬▬▬▬▬▬▬▬▬▬▬▬▬▬▬▬▬▬▬▬▬▬▬▬▬▬▬
▬▬▬▬▬▬▬▬▬▬▬▬▬▬▬▬▬▬▬▬▬▬▬▬▬▬▬▬▬▬▬▬▬▬▬▬▬▬▬▬▬▬▬▬▬▬▬▬▬▬▬▬▬
▬▬▬▬▬▬▬▬▬▬▬▬▬▬▬▬▬▬▬▬▬▬▬▬▬▬▬▬▬▬▬▬▬▬▬▬▬▬▬▬▬▬▬▬▬▬▬▬▬▬▬▬▬
▬▬▬▬▬▬▬▬▬▬▬▬▬▬▬▬▬▬▬▬▬▬▬▬▬▬▬▬▬▬▬▬▬▬▬▬▬▬▬▬▬▬▬▬▬▬▬▬▬▬▬▬▬
▬▬▬▬▬▬▬▬▬▬▬▬▬▬▬▬▬▬▬▬▬▬▬▬▬▬▬▬▬▬▬▬▬▬▬▬▬▬▬▬▬▬▬▬▬▬▬▬▬▬▬▬▬
▬▬▬▬▬▬▬▬▬▬▬▬▬▬▬▬▬▬▬▬▬▬▬▬▬▬▬▬▬▬▬▬▬▬▬▬▬▬▬▬▬▬▬▬▬▬▬▬▬▬▬▬▬
▬▬▬▬▬▬▬▬▬▬▬▬▬▬▬▬▬▬▬▬▬▬▬▬▬▬▬▬▬▬▬▬▬▬▬▬▬▬▬▬▬▬▬▬▬▬▬▬▬▬▬▬▬
10 mins thin lines down

## Slide 4
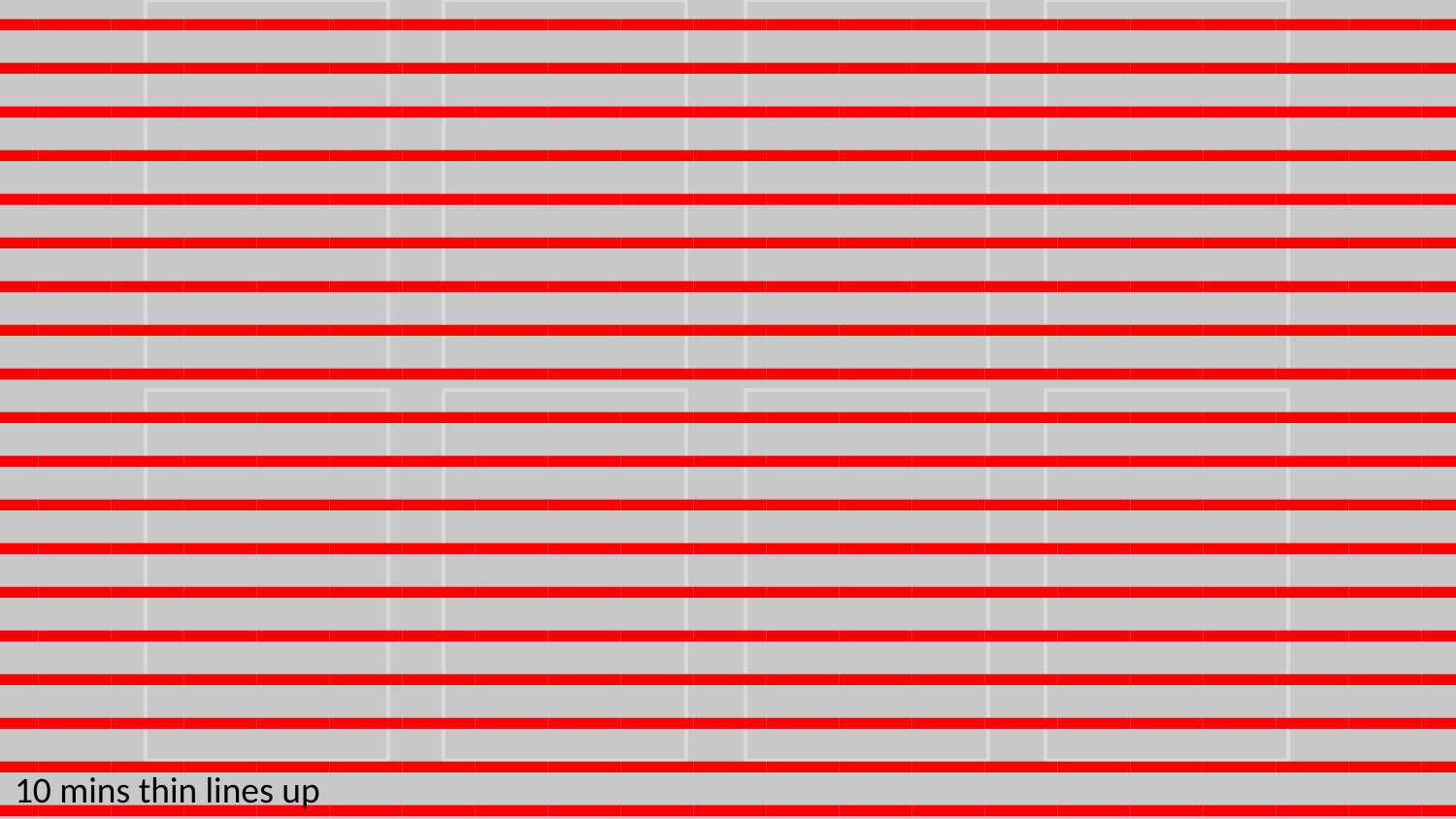

▬▬▬▬▬▬▬▬▬▬▬▬▬▬▬▬▬▬▬▬▬▬▬▬▬▬▬▬▬▬▬▬▬▬▬▬▬▬▬▬▬▬▬▬▬▬▬▬▬▬▬▬▬
▬▬▬▬▬▬▬▬▬▬▬▬▬▬▬▬▬▬▬▬▬▬▬▬▬▬▬▬▬▬▬▬▬▬▬▬▬▬▬▬▬▬▬▬▬▬▬▬▬▬▬▬▬
▬▬▬▬▬▬▬▬▬▬▬▬▬▬▬▬▬▬▬▬▬▬▬▬▬▬▬▬▬▬▬▬▬▬▬▬▬▬▬▬▬▬▬▬▬▬▬▬▬▬▬▬▬
▬▬▬▬▬▬▬▬▬▬▬▬▬▬▬▬▬▬▬▬▬▬▬▬▬▬▬▬▬▬▬▬▬▬▬▬▬▬▬▬▬▬▬▬▬▬▬▬▬▬▬▬▬
▬▬▬▬▬▬▬▬▬▬▬▬▬▬▬▬▬▬▬▬▬▬▬▬▬▬▬▬▬▬▬▬▬▬▬▬▬▬▬▬▬▬▬▬▬▬▬▬▬▬▬▬▬
▬▬▬▬▬▬▬▬▬▬▬▬▬▬▬▬▬▬▬▬▬▬▬▬▬▬▬▬▬▬▬▬▬▬▬▬▬▬▬▬▬▬▬▬▬▬▬▬▬▬▬▬▬
▬▬▬▬▬▬▬▬▬▬▬▬▬▬▬▬▬▬▬▬▬▬▬▬▬▬▬▬▬▬▬▬▬▬▬▬▬▬▬▬▬▬▬▬▬▬▬▬▬▬▬▬▬
▬▬▬▬▬▬▬▬▬▬▬▬▬▬▬▬▬▬▬▬▬▬▬▬▬▬▬▬▬▬▬▬▬▬▬▬▬▬▬▬▬▬▬▬▬▬▬▬▬▬▬▬▬
▬▬▬▬▬▬▬▬▬▬▬▬▬▬▬▬▬▬▬▬▬▬▬▬▬▬▬▬▬▬▬▬▬▬▬▬▬▬▬▬▬▬▬▬▬▬▬▬▬▬▬▬▬
▬▬▬▬▬▬▬▬▬▬▬▬▬▬▬▬▬▬▬▬▬▬▬▬▬▬▬▬▬▬▬▬▬▬▬▬▬▬▬▬▬▬▬▬▬▬▬▬▬▬▬▬▬
▬▬▬▬▬▬▬▬▬▬▬▬▬▬▬▬▬▬▬▬▬▬▬▬▬▬▬▬▬▬▬▬▬▬▬▬▬▬▬▬▬▬▬▬▬▬▬▬▬▬▬▬▬
▬▬▬▬▬▬▬▬▬▬▬▬▬▬▬▬▬▬▬▬▬▬▬▬▬▬▬▬▬▬▬▬▬▬▬▬▬▬▬▬▬▬▬▬▬▬▬▬▬▬▬▬▬
▬▬▬▬▬▬▬▬▬▬▬▬▬▬▬▬▬▬▬▬▬▬▬▬▬▬▬▬▬▬▬▬▬▬▬▬▬▬▬▬▬▬▬▬▬▬▬▬▬▬▬▬▬
▬▬▬▬▬▬▬▬▬▬▬▬▬▬▬▬▬▬▬▬▬▬▬▬▬▬▬▬▬▬▬▬▬▬▬▬▬▬▬▬▬▬▬▬▬▬▬▬▬▬▬▬▬
▬▬▬▬▬▬▬▬▬▬▬▬▬▬▬▬▬▬▬▬▬▬▬▬▬▬▬▬▬▬▬▬▬▬▬▬▬▬▬▬▬▬▬▬▬▬▬▬▬▬▬▬▬
▬▬▬▬▬▬▬▬▬▬▬▬▬▬▬▬▬▬▬▬▬▬▬▬▬▬▬▬▬▬▬▬▬▬▬▬▬▬▬▬▬▬▬▬▬▬▬▬▬▬▬▬▬
▬▬▬▬▬▬▬▬▬▬▬▬▬▬▬▬▬▬▬▬▬▬▬▬▬▬▬▬▬▬▬▬▬▬▬▬▬▬▬▬▬▬▬▬▬▬▬▬▬▬▬▬▬
▬▬▬▬▬▬▬▬▬▬▬▬▬▬▬▬▬▬▬▬▬▬▬▬▬▬▬▬▬▬▬▬▬▬▬▬▬▬▬▬▬▬▬▬▬▬▬▬▬▬▬▬▬
▬▬▬▬▬▬▬▬▬▬▬▬▬▬▬▬▬▬▬▬▬▬▬▬▬▬▬▬▬▬▬▬▬▬▬▬▬▬▬▬▬▬▬▬▬▬▬▬▬▬▬▬▬
▬▬▬▬▬▬▬▬▬▬▬▬▬▬▬▬▬▬▬▬▬▬▬▬▬▬▬▬▬▬▬▬▬▬▬▬▬▬▬▬▬▬▬▬▬▬▬▬▬▬▬▬▬
▬▬▬▬▬▬▬▬▬▬▬▬▬▬▬▬▬▬▬▬▬▬▬▬▬▬▬▬▬▬▬▬▬▬▬▬▬▬▬▬▬▬▬▬▬▬▬▬▬▬▬▬▬
10 mins thin lines up

## Slide 5
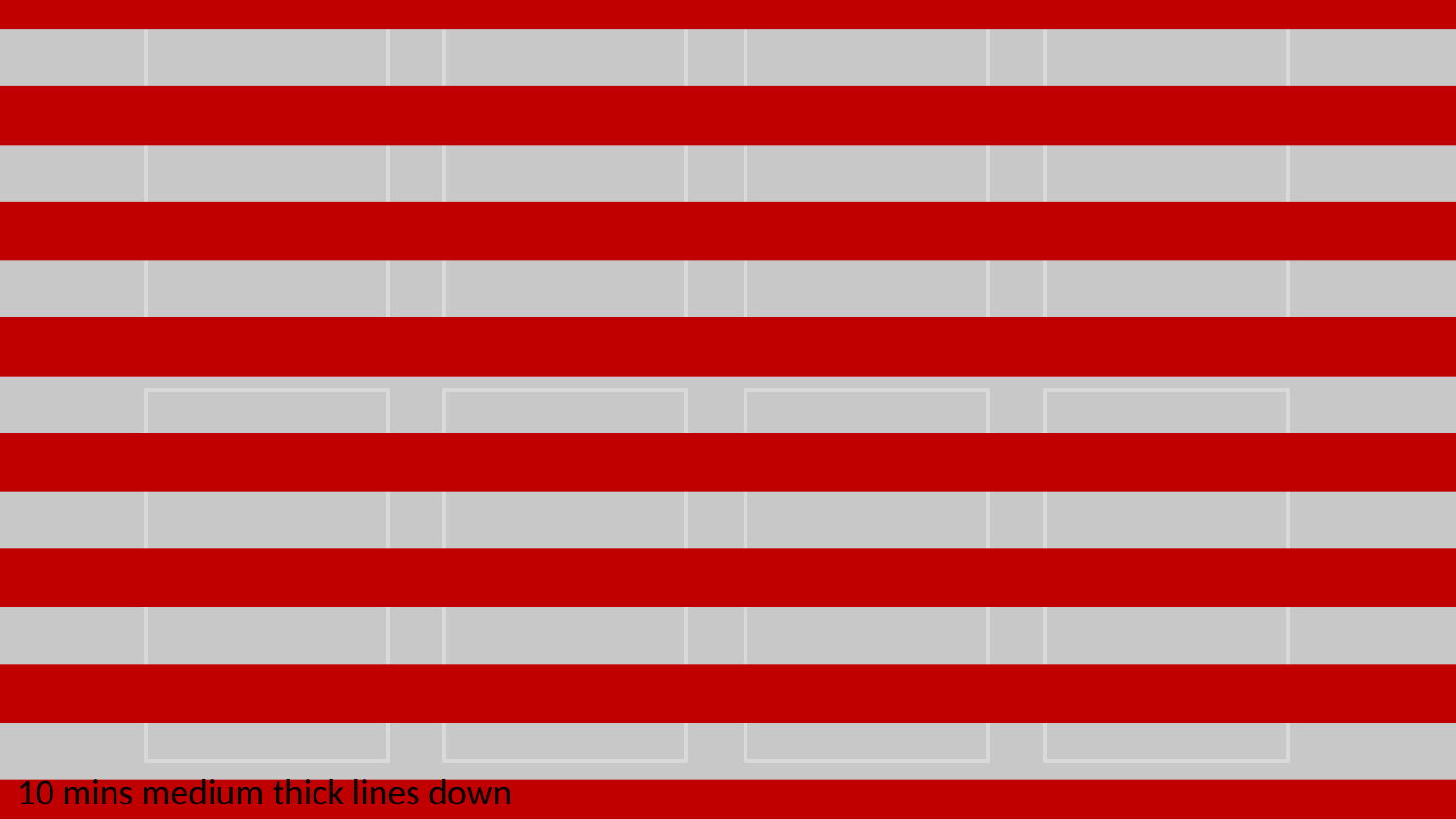

10 mins medium thick lines down

## Slide 6
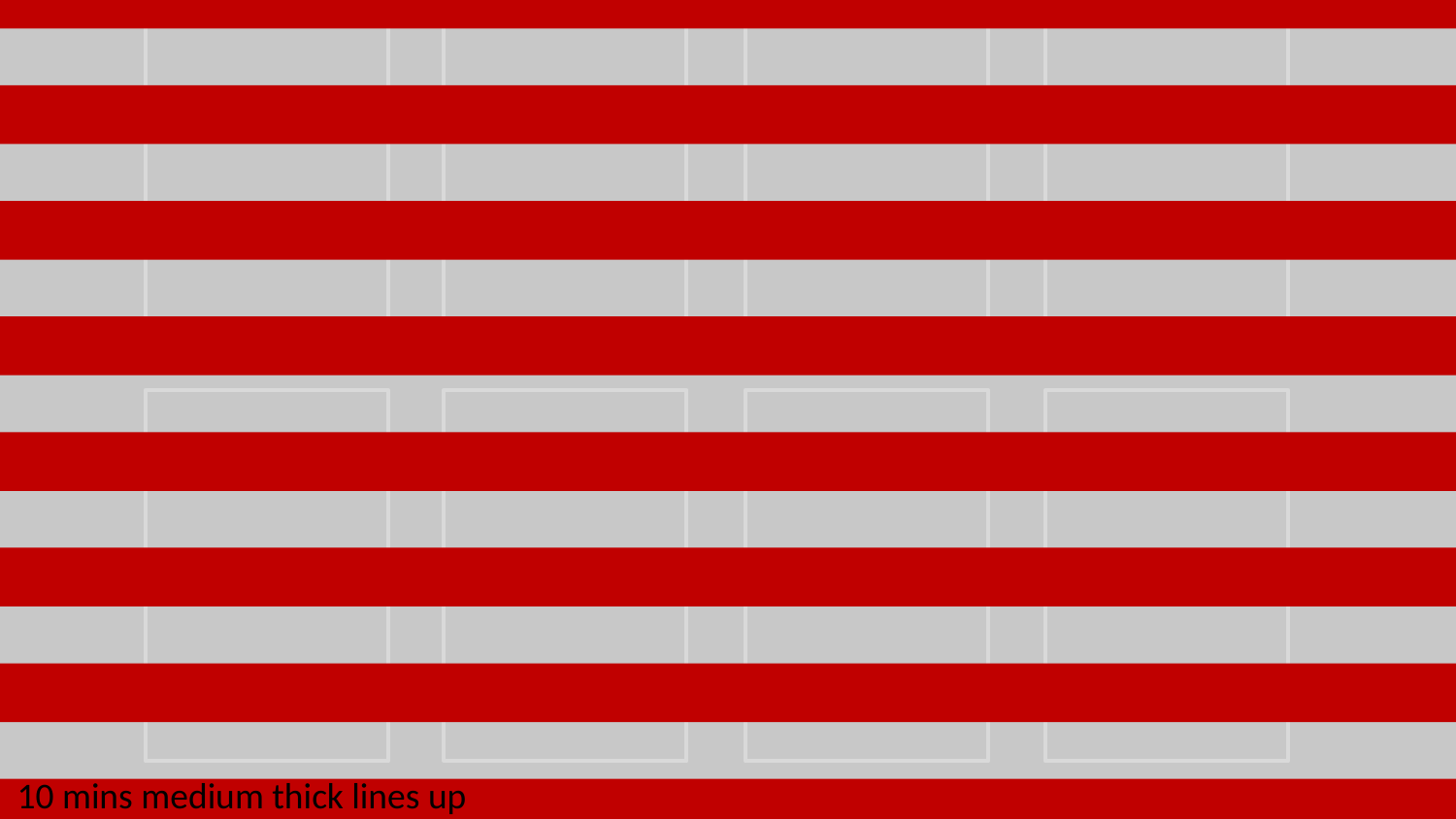

10 mins medium thick lines up

## Slide 7
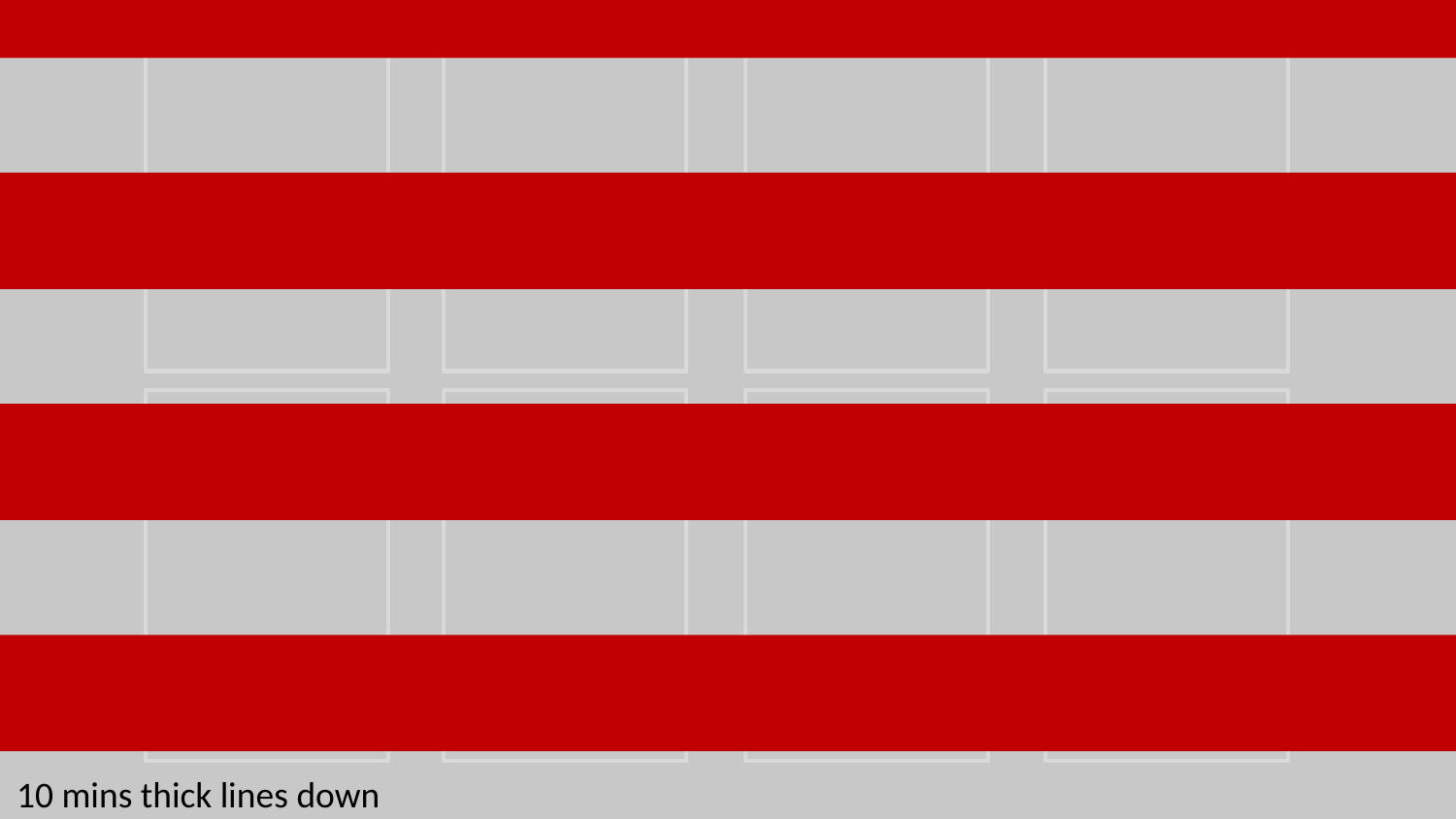

10 mins thick lines down

## Slide 8
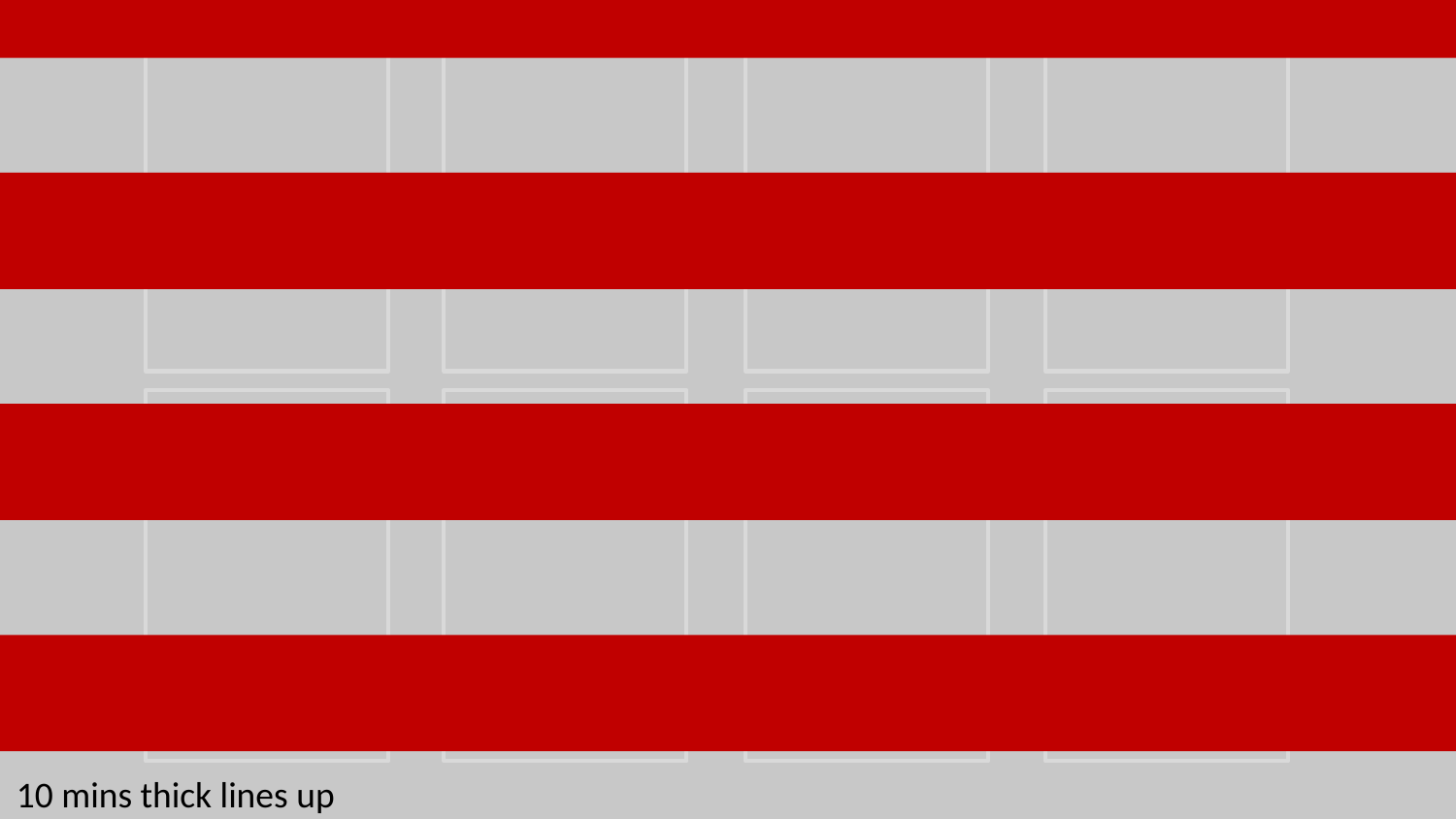

10 mins thick lines up

## Slide 9
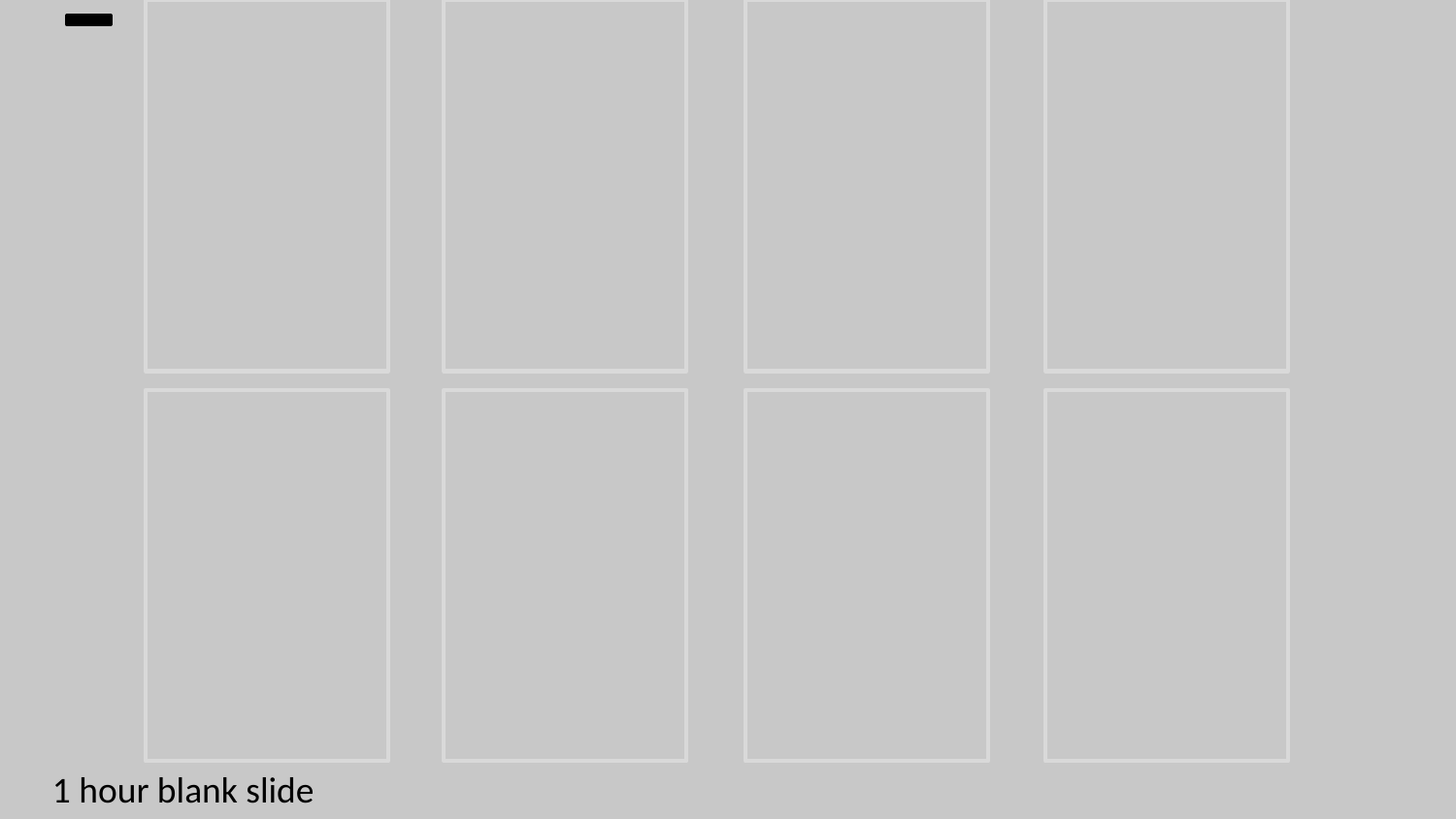

1 hour blank slide
