# Supplementary material for: Behavioral effects of visual stimuli in adult zebrafish using a novel eight-tank imaging system
Source: Front Behav Neurosci. 2024 Mar 11;18:1320126. doi: 10.3389/fnbeh.2024.1320126 (PMC10962262; doi:10.3389/fnbeh.2024.1320126)
Supplement: Supplementary file 1 [file Data_Sheet_1.ZIP › Supplementary_data_1312024/Supplementary file 7.docx]

**Supplementary file 7: Sex-specific differences in behavioral parameters across different stimuli.** The boxplots represent the median ± Interquartile range (IQR) for Move (a), End (b), OMR-Loc (c), and OMR-Or (d) for each sex (n = 8 per group) and across different visual stimuli. Blank (first hour) and Blank (third hour) were each an hour long while moving lines (left to right and right to left) were each 30 minutes long. Therefore, for each sex (n = 8), there are 48 individual data points (8 X 6 periods) for each blank (first and third hour) stimuli and 24 data points (8 X 3 periods) for moving lines (left to right and right to left) stimuli. We performed a two-way ANOVA to look at the significant effects of Sex, Stimuli, and interaction (Sex*Stimuli) for each behavioral parameter. Two-way ANOVA revealed significant differences across sex for only two of the four behavioral parameters - for End (p-value=0.01) and OMR-Or (p-value=0.038). Post-hoc pairwise comparisons were only performed for End and OMR-Or to pinpoint significant changes across sex for different stimuli. Pairwise comparisons (P-value adjustment using the Bonferroni method) showed significant sex-specific differences in the end zone for the first hour (blank) and OMR-Or for the third hour (blank). *** p-value < 0.001
