# Supplementary figures and images for: Behavioral effects of visual stimuli in adult zebrafish using a novel eight-tank imaging system
Source: Front Behav Neurosci. 2024 Mar 11;18:1320126. doi: 10.3389/fnbeh.2024.1320126 (PMC10962262; doi:10.3389/fnbeh.2024.1320126)

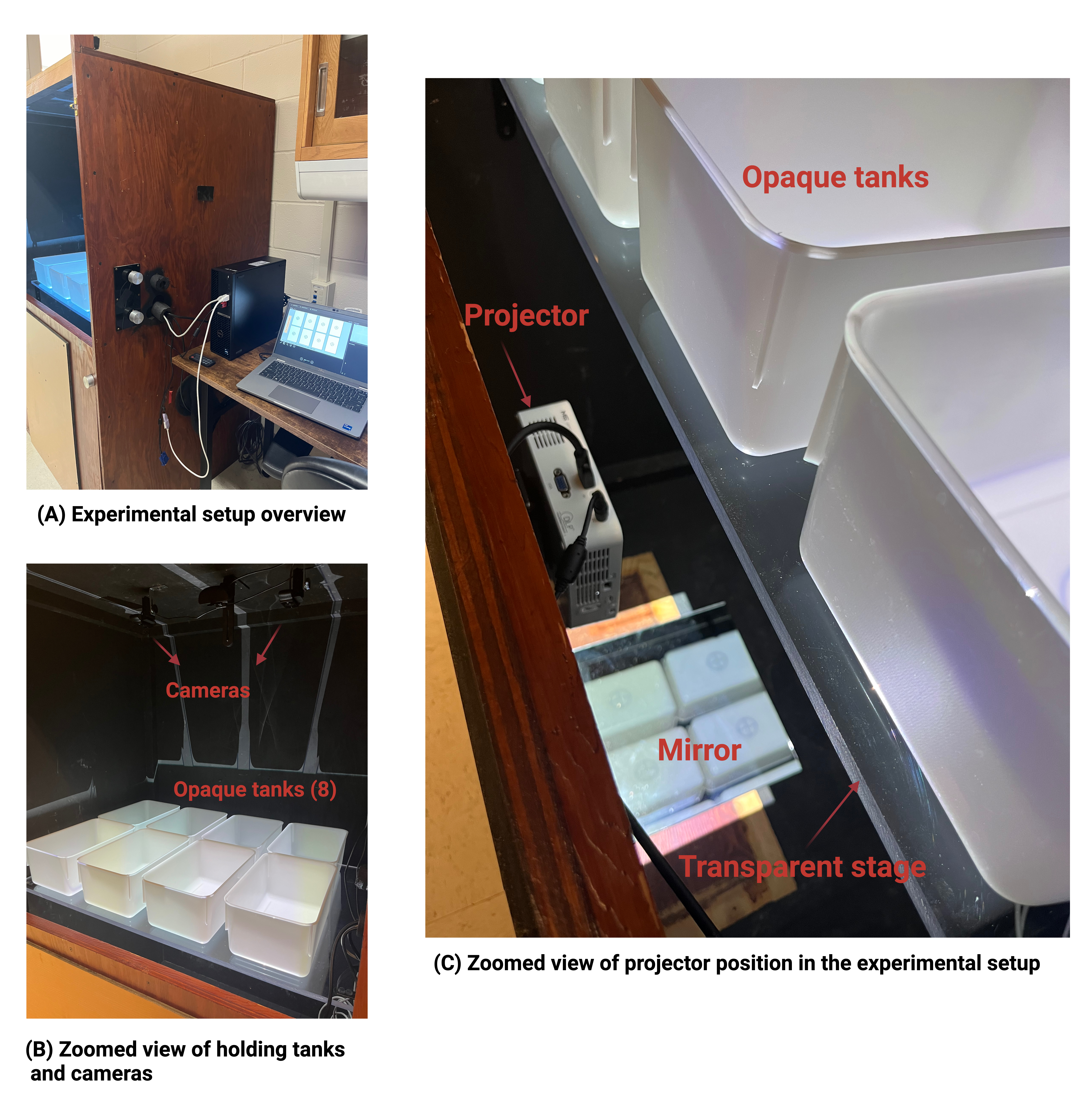

Supplement: Supplementary file 1 [file Data_Sheet_1.ZIP › Supplementary_data_1312024/Supp_file_2_setup_overview.jpg]
